# Supplementary material for: Willingness to get vaccinated initially and yearly against COVID-19 and its association with vaccine hesitancy, vaccine knowledge and psychological well-being: a cross-sectional study in UK adults
Source: BMJ Open. 2024 Jul 5;14(7):e080778. doi: 10.1136/bmjopen-2023-080778 (PMC11227763; doi:10.1136/bmjopen-2023-080778)
Supplement: Supplementary data [file bmjopen-2023-080778supp001.pdf]

Supplementary materials

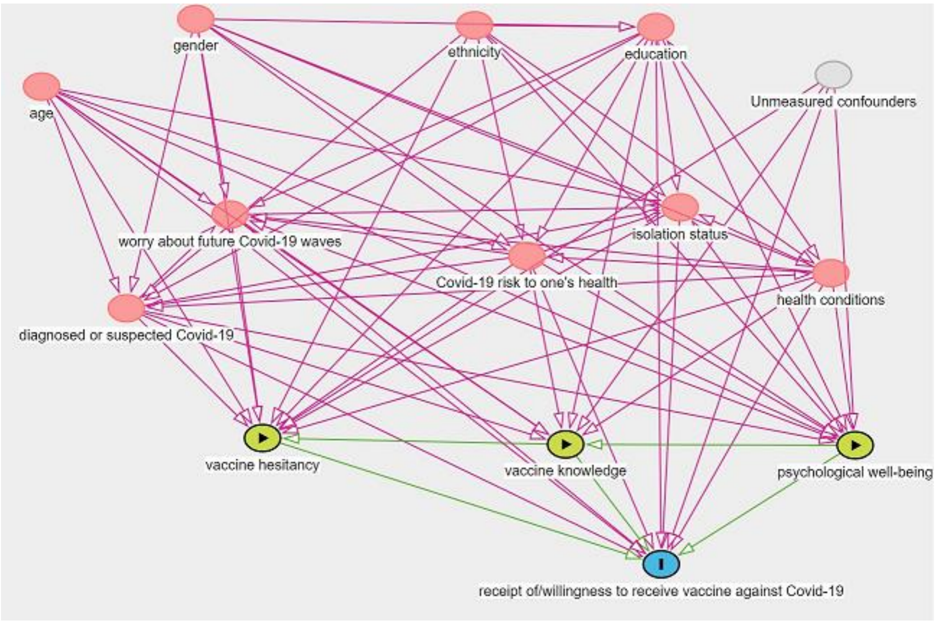

Supplementary figure 1. Directed acyclic graph (DAG) depicting which covariates should be included in a multivariable statistical model in order to minimise bias in the estimate of the total causal effect of i) vaccine hesitancy, ii) vaccine knowledge and iii) psychological well-being during the Covid-19 pandemic and (RQ1i) receipt of/willingness to receive a vaccine against Covid-19. The DAG was developed based on evidence from the existing literature and consensus within the research team.

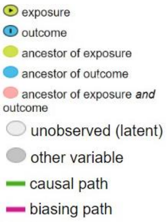

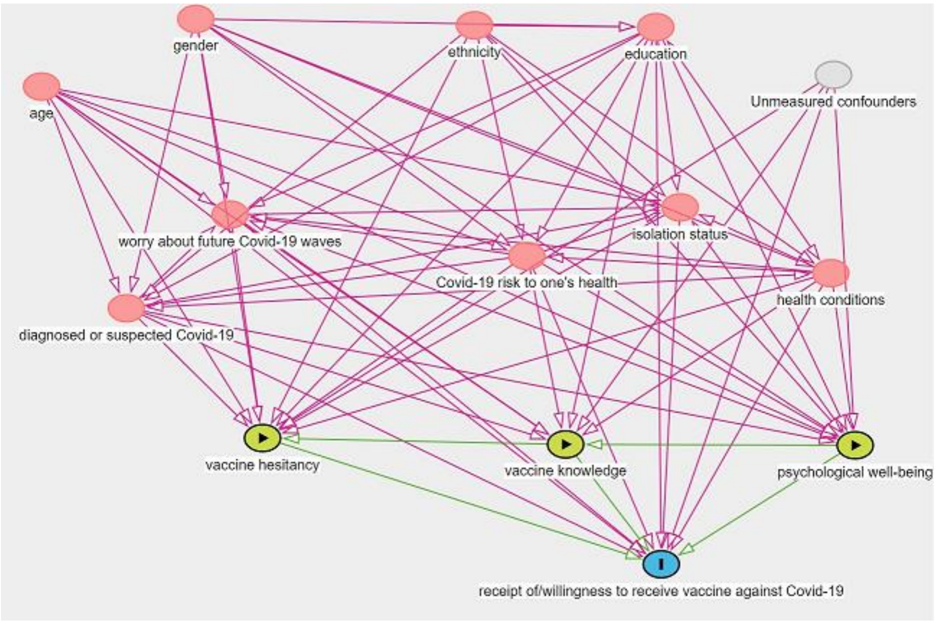

Supplementary figure 2. Directed acyclic graph (DAG) depicting which covariates should be included in a multivariable statistical model in order to minimise bias in the estimate of the total causal effect of i) vaccine hesitancy, ii) vaccine knowledge and iii) psychological well-being during the Covid-19 pandemic and (RQ2) willingness to get vaccinated against Covid-19 on a yearly basis. The DAG was developed based on evidence from the existing literature and consensus within the research team.

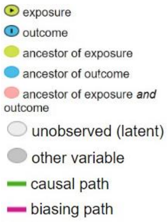

Supplementary table 1. Baseline characteristics of included and excluded sample (unweighted).

	**Total sample N=2,992**	**Included N=1,565 (52.3%)**	**Excluded N=1,427 (47.7%)**	***p***
Age in years M(SD)	47.92 (15.46)	51.74 (14.41)	43.74 (15.49)	<0.001
Female sex, % (N)	68.6 (2,054)	71.3 (1,114)	66.1 (940)	0.002
White ethnicity, % (N)	93.7 (2,804)	95.4 (1,487)	92.6 (1,317)	0.001
Post-16 qualifications, % (N)	86.7 (2,595)	88.9 (1,392)	84.3 (1,203)	<0.001
Health problems, % (N)	41.1 (1,208)	42.4 (659)	39.6 (549)	0.12

M=Mean, SD=Standard Deviation

Kale D, *et al. BMJ Open* 2024; 14:e080778. doi: 10.1136/bmjopen-2023-080778

Supplementary table 2. Associations between vaccine hesitancy and willingness to get vaccinated against Covid-19 on a yearly basis (sensitivity unweighted analysis).

	**Willingness to get yearly vaccination against Covid-19 (reference category=yes)**							
	**No**				**Not sure**			
	**N=1,565**		**N=1,406**		**N=1,565**		**N=1,406**	
	**OR [95% CI]**	***P***	**aOR [95% CI]**	***P***	**OR [95% CI]**	***P***	**aOR [95% CI]**	***P***
	**N=1,565**							
Vaccine hesitancy	147.70 [67.78-321.86]	<0.001	28.68 [14.38-57.20]	<0.001	17.89 [10.68-29.96]	<0.001	7.94 [4.48-14.08]	<0.001
Vaccine knowledge	0.27 [0.22-0.34]	<0.001	0.47 [0.36-0.62]	<0.001	0.42 [0.34-0.50]	<0.001	0.46 [0.36-0.59]	<0.001
Psychological well-being	0.69 [0.53-0.88]	0.003	1.07 [0.83-1.39]	0.596	0.74 [0.61-0.90]	0.002	0.94 [0.76-1.17]	0.580
*Health problems: yes	1 (ref)		1 (ref)		1 (ref)		1 (ref)	
No	1.30 [0.77-2.22]	0.330	1.35 [0.77-2.36]	0.290	0.85 [0.58-1.26]	0.416	0.82 [0.53-1.27]	0.372
Perceived high risk of Covid-19: no	1 (ref)		1 (ref)		1 (ref)		1 (ref)	
Yes	0.65 [0.23-1.82]	0.411	0.74 [0.28-1.94]	0.545	0.63 [0.29-1.37]	0.247	0.54 [0.24-1.20]	0.128
Confirmed/suspected Covid-19: no	1 (ref)		1 (ref)		1 (ref)		1 (ref)	
Yes	2.50 [1.49-4.17]	<0.001	1.52 [0.89-2.59]	0.122	1.61 [1.08-2.38]	0.022	1.07 [0.68-1.68]	0.773
Being in isolation: no	1 (ref)		1 (ref)		1 (ref)		1 (ref)	
Yes	4.76 [1.00-20.00]	0.049	3.60 [0.39-33.33]	0.261	1.27 [0.16-10.00]	0.821	2.21 [0.10-49.46]	0.618
**Receipt of/willingness to receive an initial Covid-19 vaccine: yes	1 (ref)		1 (ref)		1 (ref)		1 (ref)	
No	-		-		-		-	
Age (cont.)	0.99 [0.97-1.00]	0.087	0.98 [0.96-1.01]	0.123	0.98 [0.97-0.99]	0.002	0.98 [0.96-0.99]	0.005
Worry about future Covid-19 wave	0.96 [0.95-0.97]	<0.001	0.98 [0.97-0.99]	0.001	0.98 [0.97-0.99]	<0.001	1.00 [0.99-1.01]	0.436

Time of completion	1.03 [1.00-1.06]	0.032	1.04 [1.01-1.07]	0.021	1.01 [0.99-1.03]	0.480	1.00 [0.98-1.03]	0.821

OR=odds ratio; aOR=adjusted odds ratio; CI=Confidence Interval; ref=reference category
The reference category for the outcome variable was the response option 'yes'
*In unadjusted analysis of Health problems N=1,554.
** In unadjusted analysis of Receipt of/willingness to receive Covid-19 vaccine for first time N=1,415.

Kale D, et al. BMJ Open 2024; 14:e080778. doi: 10.1136/bmjopen-2023-080778
