# Supplementary material for: Willingness to get vaccinated initially and yearly against COVID-19 and its association with vaccine hesitancy, vaccine knowledge and psychological well-being: a cross-sectional study in UK adults
Source: BMJ Open. 2024 Jul 5;14(7):e080778. doi: 10.1136/bmjopen-2023-080778 (PMC11227763; doi:10.1136/bmjopen-2023-080778)
Supplement: Author's manuscript [file bmjopen-2023-080778.draft_revisions.pdf]

BMJ Open

BMJ Open is committed to open peer review. As part of this commitment we make the peer review history of every article we publish publicly available.

When an article is published we post the peer reviewers' comments and the authors' responses online. We also post the versions of the paper that were used during peer review. These are the versions that the peer review comments apply to.

The versions of the paper that follow are the versions that were submitted during the peer review process. They are not the versions of record or the final published versions. They should not be cited or distributed as the published version of this manuscript.

BMJ Open is an open access journal and the full, final, typeset and author-corrected version of record of the manuscript is available on our site with no access controls, subscription charges or pay-per-view fees (http://bmjopen.bmj.com).

If you have any questions on BMJ Open's open peer review process please email info.bmjopen@bmj.com

BMJ Open

Willingness to get vaccinated initially and yearly against Covid-19 and its association with vaccine hesitancy, vaccine knowledge and psychological well-being: a cross-sectional study in UK adults

Journal:	*BMJ Open*
Manuscript ID	bmjopen-2023-080778
Article Type:	Original research
Date Submitted by the Author:	10-Oct-2023

Complete List of Authors:	Kale, Dimitra; University College London, Department of Behavioural Science and Health; University College London Institute of Epidemiology and Health Care<br>Shoesmith, Emily; University of York<br>Herbec, Aleksandra; UCL<br>Shahab, Lion; UCL, Dept of Epidemiology and Public Health

Keywords:	Vaccination, COVID-19, PUBLIC HEALTH, EPIDEMIOLOGY

SCHOLARONE™ Manuscripts

I, the Submitting Author has the right to grant and does grant on behalf of all authors of the Work (as defined in the below author licence), an exclusive licence and/or a non-exclusive licence for contributions from authors who are: i) UK Crown employees; ii) where BMJ has agreed a CC-BY licence shall apply, and/or iii) in accordance with the terms applicable for US Federal Government officers or employees acting as part of their official duties; on a worldwide, perpetual, irrevocable, royalty-free basis to BMJ Publishing Group Ltd ("BMJ") its licensees and where the relevant Journal is co-owned by BMJ to the co-owners of the Journal, to publish the Work in this journal and any other BMJ products and to exploit all rights, as set out in our licence.

The Submitting Author accepts and understands that any supply made under these terms is made by BMJ to the Submitting Author unless you are acting as an employee on behalf of your employer or a postgraduate student of an affiliated institution which is paying any applicable article publishing charge ("APC") for Open Access articles. Where the Submitting Author wishes to make the Work available on an Open Access basis (and intends to pay the relevant APC), the terms of reuse of such Open Access shall be governed by a Creative Commons licence – details of these licences and which Creative Commons licence will apply to this Work are set out in our licence referred to above.

Other than as permitted in any relevant BMJ Author's Self Archiving Policies, I confirm this Work has not been accepted for publication elsewhere, is not being considered for publication elsewhere and does not duplicate material already published. I confirm all authors consent to publication of this Work and authorise the granting of this licence.

For peer review only

Title: Willingness to get vaccinated initially and yearly against Covid-19 and its association with vaccine hesitancy, vaccine knowledge and psychological well-being: a cross-sectional study in UK adults

Authors: Dimitra Kale[1,2], Emily Shoesmith[3], Aleksandra Herbec[1,4], Lion Shahab[1,2]

[1]Department of Behavioural Science and Health, University College London, UK

[2]SPECTRUM Consortium, UK

[3]Department of Health Sciences, University of York, York, United Kingdom

[4]Institute–European Observatory of Health Inequalities, Calisia University, Kalisz, Poland

Corresponding author: Dimitra Kale, dimitra.kale09@ucl.ac.uk

Registration: The analysis plan was pre-registered, and it is available at https://osf.io/s46qm

For peer review only - http://bmjopen.bmj.com/site/about/guidelines.xhtml

Abstract

Objectives: This study explores the association between vaccine hesitancy, vaccine knowledge and psychological well-being with (i) receipt of/willingness to receive an initial vaccine against Covid-19, and (ii) willingness to get vaccinated yearly against Covid-19. The importance of different vaccine attributes (e.g., vaccine technology, effectiveness, side effects) to choose a specific Covid-19 vaccine was also assessed.

Design: Cross-sectional survey administered during May-June 2021 on vaccine hesitancy, vaccine knowledge, psychological well-being, willingness to receive Covid-19 vaccines, sociodemographic and Covid-19-related factors.

Setting: United Kingdom.

Participants: A self-selected sample of 1,408 adults.

Results: Receipt of/willingness to receive a vaccine against Covid-19 initially and yearly were high (97.0% and 86.6%, respectively). Vaccine hesitancy as measured by a validated scale was negatively associated with receipt of/willingness to receive vaccine initially/yearly (adjusted OR (aOR)=0.09, 95%Confidence Interval (CI) 0.04-0.26, p<0.001/aOR=0.05, 95%CI 0.03-0.09; p<0.001). Vaccine knowledge and psychological well-being were positively associated with willingness to receive a yearly vaccine (aOR=1.81, 95%CI 1.43-2.29, p<0.001 and aOR=1.25, 95%CI 1.02-1.51; p=0.014; respectively) and general vaccine knowledge also with receipt of/willingness to receive vaccine initially (aOR=1.69, 95%CI 1.18-2.42; p=0.004). Vaccine effectiveness was the most important attribute for participants to choose a specific Covid-19 vaccine.

Conclusions: Improving vaccine knowledge and emphasizing vaccine efficacy may minimize vaccine hesitancy and increase Covid-19 vaccine uptake.

For peer review only - http://bmjopen.bmj.com/site/about/guidelines.xhtml

Strength and limitations of this study

- The study benefited from a large sample size.
- The use of validated measures of vaccine hesitancy and vaccine knowledge.
- Examining willingness to get vaccinated against Covid-19 during the roll-out of the first Covid-19 vaccine programme, which improves the validity of findings and minimises recall bias.
- Use of a convenience sample, which may not be representative of the general population.
- Self-reported data and thus susceptible to social desirability bias

Keywords: vaccination, vaccine hesitancy, vaccine, vaccine knowledge, psychological well-being, Covid-19, UK

For peer review only

Introduction

Covid-19, caused by SARS-CoV-2, is one of the deadliest communicable diseases of the 21st century [1], which led to a pandemic that burdened all aspects of life worldwide [2]. To contain the pandemic, vaccines against Covid-19 were quickly produced and approved for use in the general population in late 2020 and early 2021 across different countries. The development of the Covid-19 vaccines unfolded in real-time, lending a sense that they were developed more rapidly than other vaccines, some used new technology to trigger an immune response (i.e., mRNA technology), raising concerns regarding their safety and effectiveness [3]. Subsequently, these concerns have increased Covid-19 vaccine hesitation around the world [4]. According to the World Health Organization, vaccine hesitancy is the 'delay in acceptance or refusal of vaccination despite the availability of vaccination services' [5]. It is one of the 10 major threats to global health [6] and is considered one of the most disruptive factors affecting progress in vaccinating people against infectious diseases [7]. Understanding correlates of Covid-19 vaccine acceptance and vaccine attributes that are important for people when considering getting a Covid-19 vaccine (i.e., vaccine technology) is therefore critical to designing a successful immunization program that will continue to contain the Covid-19 pandemic and prevent potentially devastating outbreaks in the future. Also, it will help with the development of evidence-based interventions to address vaccine hesitancy in general, which further increases the importance of research in this area.

A growing literature studies the determinants of vaccine hesitancy related to Covid-19 and willingness to get vaccinated against Covid-19. Factors associated with willingness to get vaccinated against Covid-19 include multiple socio-demographics, behavioural and psychological correlates, as well as pandemic-related factors, such as perceived

For peer review only - http://bmjopen.bmj.com/site/about/guidelines.xhtml

susceptibility to and severity of Covid-19 [i.e., 8, 9]. Specifically, it has been shown that being older, male, of white ethnicity, with high income, and post-16 qualification have been consistently related to higher Covid-19 vaccine acceptance and lower Covid-19 vaccine hesitancy [8-12]. Additionally, a positive correlation has been reported between willingness to get vaccinated against Covid-19 and negative affect, with those reporting more worry and anxiety related to Covid-19 also reporting more willingness to get vaccinated against Covid-19 [13, 14]. However, some data suggest that poor psychological well-being and significant levels of depression are associated with vaccine hesitancy [15]. Thus, people with low levels of psychological well-being and high levels of psychological distress may have greater intolerance of uncertainty during the Covid-19 pandemic [16], and therefore may have greater fears of adverse effects and concerns about the vaccine failing to prevent Covid-19. Pandemic experiences, such as history of Covid-19 infection, higher perceived likelihood of getting Covid-19, and higher perceived severity of Covid-19, have also been associated with higher Covid-19 vaccine acceptance [11-12, 17-18]. Vaccine-related attributes such as efficacy and side effects have also been associated with willingness to receive a Covid-19 vaccination [19]. However, most of these studies were assessing hypothetical vaccine acceptance and hesitancy.

Most Covid-19 vaccinations regimes require at least two doses to reach the desired immunization level, and additionally many countries encouraged and facilitated booster doses among its population [20]. Examining rates of Covid-19 vaccination, it seems that they were higher for the first dose of Covid-19 vaccine in comparison with follow-up/booster doses. For example, in the UK the prevalence of receipt of the first Covid-19 vaccination dose was 93.6%, while 88.3% of people of all ages received the second dose and 70.2% the

For peer review only - http://bmjopen.bmj.com/site/about/guidelines.xhtml

third dose [21]. These rates confirm earlier research, suggesting that a considerable proportion of the general population who have received two doses of a Covid-19 vaccine were either unwilling or unsure about accepting a booster vaccine [14]. Factors associated with this unwillingness and uncertainty include lower levels of stress about catching or becoming seriously ill from Covid-19, lower levels of educational qualification, lower socio-economic position, and younger age [14].

For peer review only

Emerging data also consistently show that vaccine effectiveness against Covid-19 infection, both mild and severe, declines over time [22]. Thus, administering additional Covid-19 vaccine doses to appropriate individuals (i.e., elderly, those with vulnerabilities, and healthcare staff and professionals that are working with vulnerable populations) regularly may be necessary to protect susceptible individuals against hospitalisation and death. Indeed, similar to flu vaccines, the Covid-19 vaccine autumn boosters have been offered in UK to vulnerable groups and healthcare professionals. Officials in the US and UK have suggested that we will need to include annual Covid-19 vaccinations, with a schedule similar to flu shots to prevent potentially devastating outbreaks in the future [21, 23]. Crucially, the effectiveness of such programmes depends on vaccine uptake.

While several studies have sought to identify factors associated with willingness to get vaccinated against Covid-19, research examining people's willingness to get vaccinated against Covid-19 on a yearly basis is scarce. For example, Pal et al. [24] examined attitudes towards a hypothetical annual booster vaccine against Covid -19 in US healthcare workers, and they reported that overall acceptance was 83.6%. However, this acceptance was widely divergent among the vaccine-hesitant and non-hesitant group (13.8% versus 89.9%).

For peer review only - http://bmjopen.bmj.com/site/about/guidelines.xhtml

Moreover, most studies were conducted prior to the approval of a Covid-19 vaccine. It is not known whether these factors have the same importance when the outcome is real rather than hypothetical. Data for the current study were collected during the first Covid-19 vaccine roll-out (May-June 2021), and therefore our participants were asked about their willingness or acceptance of the initial Covid-19 vaccine, and willingness of a yearly Covid-19 vaccination while they had been already offered the first dose of Covid-19 vaccine or were likely to be offered it soon.

The aim of this study is to explore whether there is an association between vaccine hesitancy, general vaccine knowledge (e.g., the process related to vaccination, the impact of vaccination) and psychological well-being during the Covid-19 pandemic with (i) receipt of/willingness to receive vaccine against Covid-19 for the first time, and (ii) willingness to get vaccinated against Covid-19 on a yearly basis in a sample of UK adults during the period of the first Covid-19 vaccine roll-out. Greater understanding of the associations between vaccine hesitancy, vaccine knowledge, and psychological well-being with willingness to get vaccinated against Covid-19 could help us design mitigating strategies or interventions going forward (i.e., educational, or psychological support). We also assess to what extent different vaccine attributes (i.e., vaccine technology, effectiveness, side effects) are important for people to choose a specific Covid-19 vaccine, if they had the choice, which may also indicate attributes that are important for getting a vaccine against Covid-19.

Research questions:

RQ1: Is there an association between i)vaccine hesitancy, ii)vaccine knowledge and iii)psychological well-being during the Covid-19 pandemic with receipt of/willingness to

For peer review only - http://bmjopen.bmj.com/site/about/guidelines.xhtml

receive an initial vaccine against Covid-19 in a sample of UK adults during the period of the first Covid-19 vaccine roll-out, adjusting for relevant covariables?

RQ2: Is there an association between i)vaccine hesitancy, ii)vaccine knowledge and iii)psychological well-being during the Covid-19 pandemic with willingness to get vaccinated against Covid-19 on a yearly basis in a sample of UK adults during the period of the first Covid-19 vaccine roll-out, adjusting for relevant covariables?

RQ3: Which attributes (i.e., vaccine technology, effectiveness, side effects) are most important to people when considering getting a Covid-19 vaccine, if they had the choice?

For peer review only

Methods

Study design

Analysis of cross-sectional data of a longitudinal online survey of adults residing in the UK; the HEalth BEhaviours during the Covid-19 pandemic (HEBECO) study (https://osf.io/sbgru/). The study was approved by the Ethics Committee at the UCL Division of Psychology and Language Sciences (CEHP/2020/759). Baseline data collection occurred between April-June 2020, and follow-up surveys were administered at 1 month, 3, 6 and 12 months from the baseline participation date. The current study uses data from the baseline and 12-month follow-up surveys.

Study sample

A self-selected sample of UK–based adult (18+ years) who completed both the baseline survey of the HEBECO study between 23/04/2020 (initiation of recruitment) and 14/06/2020 (inclusive; marking the end of the first national UK lockdown), and a 12-month follow-up survey collected between May-June 2021. At the time of the 12-month follow-up

For peer review only - http://bmjopen.bmj.com/site/about/guidelines.xhtml

survey, all clinically extremely vulnerable individuals and those aged +40 had been offered at least one dose of Covid-19 vaccine in UK.

Initial recruitment into the baseline survey was online and involved sharing study invitations via multiple channels, including unpaid and paid advertisements on social media (e.g., Facebook), an email campaign across the network of UCL, other universities in UK, Public Health England, Cancer Research UK, charities, and local authorities across the UK. The full recruitment strategy is available online (https://osf.io/sbgru/).

Participants gave their consent prior to data collection. Data were captured and managed by the REDCap electronic data system [25, 26]. Participants were followed-up at 12 months after their baseline survey via email (except for participants that explicitly opted out), with up to three reminders to complete the survey sent at 12-month follow-up.

Measures

The questionnaires were developed by a multi-disciplinary team of academics with input from Cancer Research UK and Public Health England (https://osf.io/sbgru/). The questionnaires included validated items and new items based on expert consensus relating to emerging Covid-19-related aspects, as detailed below. All measures were self-reported.

Outcomes (assessed at 12-month follow-up)

Receipt of/willingness to receive vaccine against Covid-19 for first time. As Covid-19 vaccines became available in mid-December 2020 in UK, at 12-month follow-up (May-June 2021), individuals reported whether they had been offered a vaccine for Covid-19 (yes/no).

For peer review only - http://bmjopen.bmj.com/site/about/guidelines.xhtml

Those who replied yes, were asked if they had received a Covid-19 vaccine with the answer option: i)yes, both doses, ii)yes, one dose, iii)no, but I am scheduled to receive the vaccine, iv)no, I decided not to get vaccinated, v)prefer not to say. Participants were classified as willing to receive/received the vaccination if replied (i), (ii) or (iii), and not willing if they selected (iv) and (v). This measure was structured by researchers and was based on previous assessments of Covid-19 vaccine [i.e., 27].

Willingness to get vaccinated against Covid-19 on a yearly basis assessed with a question 'would you be willing to get vaccinated against the novel coronavirus (SARS-CoV-2) on a yearly basis?', with the answer options i)yes, ii)no, iii)not sure. Willingness to get vaccinated against Covid-19 on a yearly basis was conceptualised as a binary variable, yes vs no/not sure. We also ran sensitivity analysis where willingness was treated as a 3-category variable yes/no/not sure.

Importance of Covid-19 vaccine attributes was assessed by asking participants to rank in order of importance for them, if they had the choice to select a specific Covid-19 vaccine, the following six attributes; i)vaccine technology (established vs novel), ii)level of protection (how effective it is), iii)side effect profile (how easy it is tolerated), iv)delivery method (injected or not, one dose or more), v)ease of access (walk-in centres vs GP appointment), vi)reputation (manufacturer, media coverage). Participants could rank the attributes from 1=most important to 6=least important, and they could not assign the same ranking to more than one attribute.

Explanatory variables (assessed at 12-month follow-up):

For peer review only - http://bmjopen.bmj.com/site/about/guidelines.xhtml

Vaccine hesitancy was assessed using a nine-item scale [28], revised from the ten-item vaccine hesitancy survey tool developed by the SAGE working group on vaccine hesitancy [29]. The original scale assesses parental attitudes about childhood vaccines, while this adapted version focused on the respondents themselves and is validated in the UK population [29]. Participants were asked to answer nine questions related to their confidence in vaccines in general, rather than vaccines for Covid-19 specifically, on a five-point Likert scale (1=strongly agree to 5=strongly disagree). A continuous score was calculated, with higher scores reflecting a higher level of vaccine hesitancy.

Knowledge about vaccines was assessed using a nine-item scale that includes questions about the immunization process related to vaccination, the impact of vaccination and the consequences of vaccination in general, rather than vaccines for Covid-19 specifically. This scale has shown good psychometric properties [30]. Answer options were dichotomised into 1=correct, and 0=incorrect/don't know. A continuous score was calculated, with higher scores presenting a higher knowledge about vaccines.

Psychological well-being during the Covid-19 pandemic was assessed with the question 'How would you rate psychological well-being' measured on a scale from 'poor=1' to 'excellent=5'. It was treated as a continuous variable with higher scores presenting better psychological well-being [31].

Covariables

Sociodemographic characteristics were assessed at baseline and included age (continuous in years), gender (female vs all other), education (post-16 qualification vs not), ethnicity (any

For peer review only - http://bmjopen.bmj.com/site/about/guidelines.xhtml

white ethnicity vs all other/prefer not to say), health conditions (any health condition vs none/prefer not to say).

Covid-19-related characteristics were assessed at 12-month follow-up and include Covid-19 risk to one's health (major risk/significant vs moderate/minor/no risk at all/don't know), isolation status (total vs some/general/no isolation), diagnosed/suspected Covid-19 (yes vs no/prefer not to say), worry about future Covid-19 waves (continuous; 0=not at all, 50=neutral, 100=extremely, higher scores representing more worry).

Confounder variable

Timing (day) of the 12-month follow-up survey completion (daily basis; continuous) was included to control for changes in willingness to get vaccinated in light of the changes in social distancing measures in UK during the period of data collection.

Statistical analysis

Descriptive statistics were calculated to characterise the sample. Data were analysed in SPSS version 27 on complete cases only. The protocol and analysis plan were pre-registered on Open Science Framework (https://osf.io/s46qm). To account for the non-random nature of the sampling, data were weighted to the proportions of sex, age, ethnicity, education, and country of living obtained from the Office for National Statistics (2018) for the descriptive analysis. Data were unweighted for the inferential analysis.

Logistic regressions for unweighted data were conducted to examine the association between i)vaccine hesitancy, ii)vaccine knowledge and iii)psychological well-being during

For peer review only - http://bmjopen.bmj.com/site/about/guidelines.xhtml

the Covid-19 pandemic and (RQ1) receipt of/willingness to receive an initial vaccine against Covid-19, and (RQ2) willingness to get vaccinated against Covid-19 on a yearly basis. We run both unadjusted analysis and adjusted analysis controlling for relevant covariables and timing of 12-month follow-up survey. To identify the relevant covariables for each model, we drew directed acyclic graphs (DAGs) to make the assumptions about the relationships between variables explicit [32]. We used an online tool, DAGitty v3.0 to create the DAGs (http://www.dagitty.net/). Variables and their measurements are depicted by nodes and connected by unidirectional arcs depicting the hypothesised relationship. For RQ1 models, our DAG (supplementary figure 1) implied that the following confounding variables required conditioning (as a minimal sufficient adjustment set): Covid-19 risk to one's health, age, diagnosed or suspected Covid-19, education, health conditions, isolation status, worry about future Covid-19 waves. For RQ2 models, our DAG (supplementary figure 2) implied that the following confounding variables required conditioning (as a minimal sufficient adjustment set): Covid-19 risk to one's health, age, diagnosed or suspected Covid-19, health conditions, isolation status, receipt of/willingness to receive vaccine against Covid-19 for first time, worry about future Covid-19 waves.

In the sensitivity analysis, multinomial regressions were conducted to examine the association between (i)vaccine hesitancy, (ii)vaccine knowledge and (iii)psychological well-being during the Covid-19 pandemic and willingness to get vaccinated against Covid-19 on a yearly basis, where willingness was treated as a 3-category variable (i)yes, (ii)no, (iii)not sure. The reference category for the outcome variable was the response option ‘yes’.

For peer review only - http://bmjopen.bmj.com/site/about/guidelines.xhtml

For RQ3, descriptive analysis of the proportions and 95% Confidence Intervals (CI) of the most important vaccine attributes for people to choose a specific Covid-19 vaccine was conducted using weighted data.

Results

Out of a total of 2,992 UK adults recruited into the HEBECO baseline survey, 1,565 (weighted 1,408) participants were successfully followed-up at 12 months and provided data on the variables of interest, and hence met the inclusion criteria for the present analysis. Comparison of those included in the present analysis and those meeting criteria for exclusion showed that included participants were more likely to be older, female, of white ethnicity and have post-16 qualifications (Supplementary Table 1). The weighted characteristics of the included sample are presented in Table 1. Included participants were on average in their fifties, half of them were female, most of them were of white ethnicity and two thirds had post-16 qualifications. Just under half had a health problem, most had low risk perceptions for Covid-19, two thirds were worried about future Covid-19 waves, only 17 were in total isolation, and one third reported diagnosed/suspected Covid-19. The sample had low vaccine hesitancy, high vaccine knowledge and their psychological well-being was average.

Results RQ1

Of the 1,408 participants included in the present study, 87.2% (95%CI 85.3-89.0) had been offered a vaccine for Covid-19 by the time of the 12-month survey and of these, 97.0% (95%CI 96.0-98.0) had received or they were willing to receive the initial vaccine against Covid-19.

For peer review only - http://bmjopen.bmj.com/site/about/guidelines.xhtml

Both unadjusted and adjusted analyses showed a significant negative association between vaccine hesitancy and receipt of/willingness to receive an initial vaccine against Covid-19 (odds ratio (OR)=0.05, 95%CI 0.03-0.10, p<0.001; adjusted OR (aOR)=0.09, 95%CI 0.04-0.26, p<0.001, Table 2). A significant positive association was found between vaccine knowledge and receipt of/willingness to receive a vaccine against Covid-19 for first time (OR=2.71, 95%CI 2.17-3.39, p<0.001; aOR=1.69, 95%CI 1.18-2.42, p=0.004). No significant association was observed between psychological well-being during the Covid-19 pandemic and receipt of/willingness to receive an initial vaccine against Covid-19. Being older and higher levels of worry about future Covid-19 waves were significantly associated with receipt of/willingness to receive an initial vaccine against Covid-19 (Table 2).

Results RQ2

Of the 1,408 participants, 86.6% (95%CI 84.7-88.5) were willing to get vaccinated against Covid-19 on a yearly basis. Both unadjusted and adjusted analyses showed a significant negative association between vaccine hesitancy (OR=0.03, 95%CI 0.02-0.05, p<0.001; aOR=0.05, 95%CI 0.03-0.09; p<0.001) and willingness to get vaccinated against Covid-19 on a yearly basis. Willingness to get yearly vaccinations against Covid-19 was positively associated with vaccine knowledge (OR=2.80, 95%CI 2.37-3.32, p<0.001; aOR=1.81, 95%CI 1.43-2.29; p<0.001) and psychological well-being (OR=1.39, 95%CI 1.19-1.62, p<0.001; aOR=1.25, 95%CI 1.02-1.51; p=0.014, Table 3). Being older, higher levels of worry about future Covid-19 waves, and receipt of/willingness to receive an initial Covid-19 vaccine were significantly associated with willingness to get yearly vaccination against Covid-19 (Table 3).

For peer review only - http://bmjopen.bmj.com/site/about/guidelines.xhtml

Sensitivity analysis confirmed results, finding a significant negative association between vaccine hesitancy and willingness to get vaccinated against Covid-19 on a yearly basis (Supplementary Table 1). Similarly, there was a significant positive association between vaccine knowledge and psychological well-being with willingness to get yearly vaccines against Covid-19 (Supplementary Table 1).

Results RQ3

By far the most important vaccine attribute for participants to choose a specific Covid-19 vaccine was effectiveness (57.2%, 95%CI 54.5-59.8), while the least important was related to reputation of the vaccine based on the manufacturer and media coverage (9.7%, 95%CI 8.2-11.3). All other vaccine attributes were rated as similarly unimportant; vaccine technology: 13.3%, 95%CI 11.6-15.2; side-effects: 13.6%, 95%CI 11.9-15.5; delivery method: 12.4%, 95%CI 10.7-14.2; and ease of access: 13.4% 95%CI 11.6-15.2.

Discussion

In this sample of UK adults, vaccine hesitancy was negatively associated with receipt of/willingness to receive a first vaccine against Covid-19 and willingness to get a yearly Covid-19 vaccine. Vaccine knowledge was positively associated with receipt of/willingness to receive the initial vaccine against Covid-19 and willingness to get a yearly Covid-19 vaccine, while there was also a positive association between psychological well-being during the Covid-19 pandemic and willingness to get a yearly Covid-19 vaccine only. Effectiveness of the vaccine was the most important attribute for participants to choose a specific Covid-19 vaccine if they had the choice, while the least important was reputation of the vaccine based on the manufacturer and media coverage.

For peer review only - http://bmjopen.bmj.com/site/about/guidelines.xhtml

It should be noted that in the current study an overwhelming majority of respondents had either received or were willing to receive the first dose of Covid-19 vaccine, while vaccine hesitancy was low. Previous studies conducted in the UK have documented higher vaccine hesitancy and lower vaccine acceptance rates (between 63% - 69%; e.g. [33, 34]). However, most previous work was carried out in the early days of the Covid-19 vaccine roll out, while our data were collected more than half-way through the vaccination programme in the UK. At that point of data collection, people might have had more confidence in the safety and effectiveness of Covid-19 vaccine, since not many side effects were reported from individuals who had already received the vaccine. It was also a period when Covid-19 measures were favourable for people who were vaccinated (i.e., fully vaccinated did not have to quarantine when travelling back to England), which might have also influenced individuals' decision to get vaccinated in order to enable them to resume travelling and other activities and return to 'normal living' after a long period of social distancing measures and quarantines. Longitudinal and cross-sectional studies also have found that Covid-19 vaccination intention has increased over time in the UK [9, 35], with high compliance rates [21].

The present study's high percentage of Covid-19 vaccine acceptance might also be attributed to the sociodemographic characteristics of the participants. For example, vaccine acceptance has been linked with being older, male, of white ethnicity, and having higher qualification [i.e., 8-10], which reflects our sample characteristics. However, it should be noted that we weighted the sample to be representative of the population, so this cannot entirely explain the high acceptance rates observed.

For peer review only - http://bmjopen.bmj.com/site/about/guidelines.xhtml

For peer review only

Willingness to get vaccinated against Covid-19 on a yearly basis was also high, but lower than for the first dose of Covid-19 vaccine, and similar to previous research on US healthcare workers, which suggested an acceptance rate of a hypothetical annual booster vaccine against Covid -19 of 83.6% [24]. The lower levels of willingness to get a yearly Covid-19 vaccine compared with the first dose might be attributed to the fact that participants were less worried about a future wave of Covid-19, thus assuming they will not need protection against Covid-19 provided by the vaccine. Additionally, since people know that boosters are reserved for special populations (i.e., all adults aged 50 years and over, healthcare professionals, and those with a clinical risk [21], they may think yearly vaccinations are unnecessary for themselves.

Vaccine knowledge was high in our sample, and it was positively associated with willingness to get vaccinated against Covid-19 initially and on a yearly basis. This may suggest that individuals who are better informed about vaccines in general have a greater understanding of the risk-benefit calculus of vaccinations, and thus of the benefits of the Covid-19 vaccine, specifically, for individual and population health. Additionally, most of the participants had post-16 qualifications, and it has been documented that participant with a bachelor degree are more likely to have a higher health literacy leading to a greater understanding of vaccine development and clinical trials to stop the spread of disease [36]. Earlier research also suggests that insufficient knowledge about the new Covid-19 vaccine and fears of long-term side effects were reasons not to get vaccinated. However, as more knowledge about the vaccine accumulated, and people become more educated about the vaccines, this may have increases willingness to accept the Covid-19 vaccine [37-39].

For peer review only - http://bmjopen.bmj.com/site/about/guidelines.xhtml

Psychological well-being in our sample was average and positively associated only with willingness to get a yearly Covid-19 vaccine, and not receipt of/willingness to receive the initial Covid-19 vaccine. The average levels of psychological well-being reported by our sample may be due to the timing of data collection. High levels of psychological distress were documented during nationwide lockdowns and when infection levels and subsequent death rates were at their peak [i.e., 40]. Though our study was conducted at a time when a new phase in the UK government's response to the pandemic was underway, moving away from stringent restrictions on day-to-day lives, towards advising people on how to protect themselves and others [41] and allowing for more socialisation. Additionally, an effective vaccine had already been developed and deployed. Previous research has found conflicting results about the association between psychological well-being and receipt of Covid-19 vaccine, with some findings linking poor psychological well-being, high levels of anxiety and worry with willingness to get vaccinated against Covid-19 [13, 14], and others reporting significant association between poor psychological well-being and high levels of vaccine hesitancy [15]. Our results also suggest that people with higher levels of psychological well-being were more willing to get vaccinated against Covid-19 on a yearly basis. It could be argued that people with better psychological well-being may be less prone to worry/be anxious when it comes to trying new products/vaccine, and thus more willing to get vaccinated against Covid-19.

Consistent with previous studies [i.e., 35, 42] our findings indicate that vaccine efficacy is one of the most important determinants for Covid-19 vaccine acceptance. From a practical perspective, promotional materials could emphasize Covid-19 vaccine efficacy as a strategy

For peer review only - http://bmjopen.bmj.com/site/about/guidelines.xhtml

to enhance the willingness for Covid-19 vaccine uptake. Aside from dispelling misinformation about vaccines, it may also be helpful to provide individuals with more information on why a particular type of vaccine can be effective.

Strengths and Limitations

The study benefited from a large sample size, use of validated measures of vaccine hesitancy and vaccine knowledge, as well as from examining willingness to get vaccinated against Covid-19 during a declared pandemic, and at a time during the roll-out of the first Covid-19 vaccine programme. This improves the validity of findings and minimises recall bias as the study was conducted when vaccination uptake was being actively promoted by government and health promotion authorities, and participants were faced with the actual question on whether or not to get vaccinated.

The study also has several limitations, including the use of a convenience sample who may have participated in the study due to a higher interest in the pandemic than in the general population. This interest may also be related to a higher receipt/willingness to get vaccinated against Covid-19. Additionally, the majority of participants were of white ethnicity, which meant we could not look at other ethnicities separately, when ethnic disparities in vaccine uptake/hesitancy have been reported elsewhere [43]. All data were self-reported and thus susceptible to social desirability bias. However, our rates of receipt/willingness to receive the first dose of Covid-19 vaccine were similar to the UK government rates of the first dose of Covid-19 vaccine [21]. Additionally, receipt of/willingness to receive an initial vaccine against Covid-19 was assessed only among those

For peer review only - http://bmjopen.bmj.com/site/about/guidelines.xhtml

who have been offered a vaccine against Covid-19 at the time of the 12-month survey, thus excluding some younger participants.

Conclusions

In conclusion, our findings suggest that willingness to get vaccinated against Covid-19 in a sample of UK adults during the time of the vaccine roll out was high, vaccine hesitancy was low, general vaccine knowledge was high and psychological well-being was average. There was a negative association between vaccine hesitancy and acceptance/willingness to receive the initial dose of a Covid-19 vaccine and a yearly booster, a positive association between vaccine knowledge and willingness to receive both initial and yearly doses of a Covid-19 vaccine, while there was also a positive association between psychological well-being during the Covid-19 pandemic and willingness to get a yearly Covid-19 vaccine. Effectiveness of the vaccine was the most important attribute for participants to choose a specific Covid-19 vaccine, if they had the choice. Improving knowledge about vaccines and emphasizing vaccine efficacy could minimise vaccine hesitancy and enhance the willingness for Covid-19 vaccine uptake.

(4500 words)

Declarations

Ethics approval and consent to participate

For peer review only - http://bmjopen.bmj.com/site/about/guidelines.xhtml

The study has been approved by UCL Research Ethics Committee at the UCL Division of Psychology and Language Sciences (PaLS) (CEHP/2020/579) as part of the larger programme 'The optimisation and implementation of interventions to change behaviours related to health and the environment'. The authors assert that all procedures contributing to this work comply with the ethical standards of the relevant national and institutional committees on human experimentation and with the Helsinki Declaration of 1975, as revised in 2008. All participants provided fully informed consent. The study is GDPR compliant.

Consent for publication

Not applicable.

Availability of data

The dataset used and analysed during the current study are available from the corresponding author (Dimitra Kale) on reasonable request.

Competing Interests

DK, ES, AH declare no conflicts of interest. LS has received honoraria for talks, an unrestricted research grant and travel expenses to attend meetings and workshops from Pfizer and has acted as paid reviewer for grant awarding bodies and as a paid consultant for health care companies.

Funding

For peer review only - http://bmjopen.bmj.com/site/about/guidelines.xhtml

For peer review only

This project is partially funded by an ongoing Cancer Research UK Programme Grant to UCL Tobacco and Alcohol Research Group [PRCRPG-Nov21\100002] and by SPECTRUM a UK Prevention Research Partnership Consortium [grant number MR/S037519/1]. Funders had no role in the design and conduct of the study; collection, management, analysis and interpretation of the data; preparation, review or approval of the manuscript; and decision to submit the manuscript for publication. For the purpose of Open Access, the author has applied a CC BY public copyright licence to any Author Accepted Manuscript version arising from this submission.

Author contribution

LS, AH, and DK conceived and designed the study. DK analysed the data and wrote the first draft. LS, ES and AH provided critical revisions. All authors read and approved the submitted manuscript.

Acknowledgements

We are grateful to all participants who have been supporting our research. We would like to thank Public Health England, and particularly members of the Behavioural Insights at Public Health England for providing feedback on the survey wording. We would like to thank Public Health England, Cancer Research UK, local authorities, Mayors' offices, as well as charities and other organisations in the UK, including the Asthma UK and British Lung Foundation Partnership, for supporting our recruitment campaign.

References

For peer review only - http://bmjopen.bmj.com/site/about/guidelines.xhtml

[1]Wilder-Smith, A. 2021. COVID-19 in comparison with other emerging viral diseases: risk of geographic spread via travel. Trop Dis Travel Med Vaccines 7, 3.

[2]Keni, R., Alexander, A., Nayak, P. G., Mudgal, J., Nandakumar, K., 2020. COVID-19: Emergence, spread, possible treatments, and global burden. Frontiers in Public Health, 8. https://doi.org/10.3389/fpubh.2020.00216

[3]Chavda, V.P., Chen, Y., Dave, J., Chen, Z., Chauhan, S.C., Yallapu, M.M., Uversky, V.N., Bezbaruah, R., Patel, S., Apostolopoulos V., 2022. COVID-19 and vaccination: myths vs science, Expert Review of Vaccines, 21:11, 1603-1620, doi: 10.1080/14760584.2022.2114900

[4]Sallam, M., 2021. COVID-19 Vaccine Hesitancy Worldwide: A Concise Systematic Review of Vaccine Acceptance Rates. Vaccines, 16;9(2):160. doi: 10.3390/vaccines9020160. PMID: 33669441; PMCID: PMC7920465.

[5]MacDonald, N. E.; SAGE Working Group on Vaccine Hesitancy, 2015. Vaccine hesitancy: definition, scope and determinants. Vaccine, 33, 4161–4164

[6]World Health Organization (WHO), 2020. Ten threats to global health in 2019. https://www.who.int/news-room/spotlight/ten-threatsto-global-health-in-2019. Assessed November 2022.

[7]Geoghegan, S., O'Callaghan, K.P., Offit, P.A., 2020. Vaccine safety: myths and misinformation. Frontiers in Microbiology, 11, 372

[8]Cascini, F., Pantovic, A., Al-Ajlouni, Y., Failla, G., Ricciardi W., 2021. Attitudes, acceptance and hesitancy among the general population worldwide to receive the COVID-19 vaccines and their contributing factors: A systematic review. E Clinical Medicine. 40:101113.

For peer review only - http://bmjopen.bmj.com/site/about/guidelines.xhtml

[9]Paul, E., Steptoe, A., Fancourt, D., 2021. Attitudes towards vaccines and intention to vaccinate against COVID-19: implications for public health communications. Lancet Reg Health - Eur. 1(100012)

[10]Fisher, K.A., Bloomstone, S.J., Walder, J., Crawford, S., Fouayzi, H., Mazor, K.M., 2020. Attitudes toward a potential SARS-CoV-2 vaccine. Ann. Intern. Med. 173, 964–973

[11]Malik, A.A., McFadden, S.M., Elharake, J., Omer, S.B., 2020. Determinants of COVID-19 vaccine acceptance in the US. Clinical Medicine 26. https://doi.org/10.1016/j.eclinm.2020.100495.

[12]Reiter, P.L., Pennell, M.L., Katz, M.L., 2020. Acceptability of a COVID-19 vaccine among adults in the United States: how many people would get vaccinated? Vaccine 38, 6500–6507. https://doi.org/10.1016/j.vaccine.2020.08.043.

[13]Kejriwal, M., Shen, K., 2021. COVID-19 vaccine hesitancy is positively associated with affective wellbeing. https://doi.org/10.31234/osf.io/nkvhs

[14]Paul, E., Fancourt, D., 2022. Predictors of uncertainty and unwillingness to receive the COVID-19 booster vaccine: An observational study of 22,139 fully vaccinated adults in the UK. The Lancet Regional Health – Europe, Volume 14, 100317

[15]Sekizama, Y., Hashimoto, S., Denda, K., Ochi S., So, M., 2022. Association between COVID-19 vaccine hesitancy and generalized trust, depression, generalized anxiety and fear of COVID-19. BMC Public Health. 22:126. doi: 10.1186/s12889-021-12479-w

[16]Reizer, A., Geffen, L., Koslowsky, M., 2021. Life under the COVID-19 lockdown: On the relationship between intolerance of uncertainty and psychological distress. Psychol Trauma. 13(4):432-437. doi: 10.1037/tra0001012. Epub 2021 Feb 4. PMID: 33539162.

For peer review only

[17]Khubchandani, J., Sharma, S., Price, J.H., Wiblishauser, M.J., Sharma, M., Webb, F.J., 2021. COVID-19 vaccination hesitancy in the United States: a rapid national assessment. J. Community Health 46, 270–277. https://doi.org/10.1007/s10900-020-00958-x.

[18]Killgore, W.D.S., Cloonan, S.A., Taylor, E.C., Dailey, N.S., 2021. The COVID-19 vaccine is here—now who is willing to get it? Vaccines 9, 339. https://doi.org/10.3390/vaccines9040339.

[19]Kreps, S., Prasad, S., Brownstein, J.S., Hswen, Y., Garibaldi, B.T., Zhang, B., Kriner, D. L., 2020. Factors Associated With US Adults' Likelihood of Accepting COVID-19 Vaccination. JAMA Netw Open. 3(10):e2025594. doi:10.1001/jamanetworkopen.2020.25594

[20]World Health Organization (WHO), 2022. COVID-19 advice for the public: Getting vaccinated. https://www.who.int/emergencies/diseases/novel-coronavirus-2019/covid-19-vaccines/advice#:~:text=Getting%20a%20third%20dose%20can,when%20it%20is%20your%20turn.&text=It%20is%20safe%20for%20most,get%20vaccinated%20against%20COVID%2D19. Accessed December 2022.

[21]GOV.UK, 2022. Vaccinations in United Kingdom. https://coronavirus.data.gov.uk/details/vaccinations. Accessed November 2022

[22]Ferdinands, J.M., Rao, S., Dixon, B.E., Mitchell, P.K., DeSilva, M.B., Irving, S.A. et al., 2022. Waning of vaccine effectiveness against moderate and severe covid-19 among adults in the US from the VISION network: test negative, case-control study BMJ, 379 :e072141, doi:10.1136/bmj-2022-072141

For peer review only

For peer review only - http://bmjopen.bmj.com/site/about/guidelines.xhtml

[23]Public Health Agency, 2022. COVID-19 vaccination - A guide to the autumn booster. https://www.publichealth.hscni.net/publications/covid-19-vaccination-guide-autumn-booster. Accessed November 2022.

[24]Pal, S., Shekhar, R., Kottewar, S., Upadhyay, S., Singh, M., Pathak, D., Kapuria, D., Barrett, E., Sheikh, A.B., 2021. COVID-19 Vaccine Hesitancy and Attitude toward Booster Doses among US Healthcare Workers. Vaccines 2021, 9, 1358. https://doi.org/10.3390/vaccines9111358

[25]Harris, P.A., Taylor, R., Thielke, R., Payne, J., Gonzalez, N., Conde, J.G., 2009. Research electronic data capture (REDCap)—A metadata driven methodology and workflow process for providing translational research informatics support. J. Biomed. Inform. 42, 377–381.

[26]Harris, P.A., Taylor, R., Minor, B.L., Elliott, V., Fernandez, M., O'Neal, L., McLeod, L., Delacqua, G., Delacqua, F., Kirby, J., 2019. The REDCap consortium: Building an international community of software platform partners. J. Biomed. Inform. 95, 103208.

[27]Household Pulse Survey, 2021. Measuring household experiences during the coronavirus pandemic. Census.gov. https://www.census.gov/data/experimental-data-products/household-pulse-survey.html

[28]Luyten, J., Bruyneel, L., van Hoek, A.J., 2019. Assessing vaccine hesitancy in the UK population using a generalized vaccine hesitancy survey instrument. Vaccine. 24;37(18):2494-2501. doi: 10.1016/j.vaccine.2019.03.041

[29]Larson, H. J., Jarrett, C., Schulz, W. S., Chaudhuri, M., Zhou,Y., Dube, E. et al.; SAGE Working Group on Vaccine Hesitancy, 2015. Measuring vaccine hesitancy: the development of a survey tool. Vaccine, 33, 4165–4175

For peer review only - http://bmjopen.bmj.com/site/about/guidelines.xhtml

[30]Zingg, A., Siegrist, M., 2012. Measuring people's knowledge about vaccination: Developing a one-dimensional scale. Vaccine. 30:3771-3777. https://doi.org/10.1016/j.vaccine.2012.03.014

[31]Dicken, S.J., Mitchell, J.J., Newberry Le Vay, J., Beard, E., Kale, D., Herbec, A., Shahab, L., 2021. Impact of COVID-19 pandemic on weight and BMI among UK adults: a longitudinal analysis of data from the HEBECO study. Nutrients. 2021; 13(9):2911, doi:10.3390/nu13092911

[32]Tennant, P.W.G., Murray, E.J., Arnold, K.F., Berrie, L., Fox, M.P., Gadd, S.C., et al., 2021. Use of directed acyclic graphs (DAGs) to identify confounders in applied health research: review and recommendations. Int J Epidemiol. 50(2):620–32.

[33]Murphy, J., Vallières, F., Bentall, R.P., Shevlin, M., McBride, O., Hartman, T.K., McKay, R., Bennett, K., Mason, L., Gibson-Miller, J., Levita, L., Martinez, A.P., Stocks, T.V.A., Karatzias, T., Hyland, P., 2021.. Psychological characteristics associated with COVID-19 vaccine hesitancy and resistance in Ireland and the United Kingdom. Nat Commun 12, 29 (2021). https://doi.org/10.1038/s41467-020-20226-9

[34]Sherman, S.M., Smith, L.E., Sim, J., Amlôt, R., Cutts, M., Dasch, H., Rubin, G.J., Sevdalis, N., 2021. COVID-19 vaccination intention in the UK: results from the COVID-19 vaccination acceptability study (CoVAccS), a nationally representative cross-sectional survey. Hum Vaccin Immunother. 3;17(6):1612-1621. doi: 10.1080/21645515.2020.1846397.

[35]Byrne, T., Patel, P., Shrotri, M., Beale, S., Michie, S., Butt, J., Hawkins, N., Hardelid, P., Rodger, A., Aryee, A., Braithwaite, I., Fong, W.L.E., Fragaszy, E., Geismar, C., Kovar, J., Navaratnam, A.M.D., Nguyen, V., Hayward, A., Aldridge. R.W.; Virus Watch Collaborative. 2021. Trends, patterns and psychological influences on COVID-19

For peer review only - http://bmjopen.bmj.com/site/about/guidelines.xhtml

vaccination intention: Findings from a large prospective community cohort study in England and Wales (Virus Watch). Vaccine. 26;39(48):7108-7116. doi: 10.1016/j.vaccine.2021.09.066.

[36]Sukys, S., Cesnaitiene, V.J., Ossowsky, Z.M. 2017. Is health education at university associated with students' health literacy? Evidence from cross-sectional study applying HLS-EU-Q. Biomed. Res. Int. 8516843

[37]Grech, V., Gauci, C., Agius, S. 2020. Withdrawn: Vaccine hesitancy among Maltese Healthcare workers toward influenza and novel COVID-19 vaccination. Early Hum Dev. 1:105213. doi: 10.1016/j.earlhumdev.2020.105213.

[38]Kwok, K.O., Li, K.K., Wei, W.I., Tang, A., Wong, S.Y.S., Lee, S.S. 2021. Influenza vaccine uptake, COVID-19 vaccination intention and vaccine hesitancy among nurses: A survey. International Journal of Nursing Studies, 114

[39]Rabi, R., Maraqa, B., Nazzal, Z., Zink, T. 2021. Factors affecting nurses' intention to accept the COVID-19 vaccine: A cross-sectional study. Public Health Nurs. 38(5):781-788. doi: 10.1111/phn.12907

[40]Aknin, L. B., Andretti, B., Goldszmidt, R., Helliwell, J. F., Petherick, A., De Neve, J., Dunn, E. W., Fancourt, D., Goldberg, E., Jones, S. P., Karadag, O., Karam, E., Layard, R., Saxena, S., Thornton, E., Whillans, A., Zaki, J. 2022. Policy stringency and mental health during the COVID-19 pandemic: a longitudinal analysis of data from 15 countries, The Lancet Public Health, 7(5): e417-e426

[41]GOV.UK, 2021. Guidance. COVID-19 response: Summer 2021. https://www.gov.uk/government/publications/covid-19-response-summer-2021-roadmap/covid-19-response-summer-2021. Acessed March 2023

For peer review only - http://bmjopen.bmj.com/site/about/guidelines.xhtml

[42]Freeman, D., Loe, B.S., Chadwick, A., Vaccari, C., Waite, F., Rosebrock, L., Jenner, L., Petit, A., Lewandowsky, S., Vanderslott, S., Innocenti, S., Larkin, M., Giubilini, A., Yu, L.M., McShane, H., Pollard, A.J., Lambe, S. 2022. COVID-19 vaccine hesitancy in the UK: the Oxford coronavirus explanations, attitudes, and narratives survey (Oceans) II. Psychol Med. 52(14):3127-3141. doi: 10.1017/S0033291720005188

[43]Nguyen, L.H., Joshi, A.D., Drew, D.A. et al. (2022). Self-reported COVID-19 vaccine hesitancy and uptake among participants from different racial and ethnic groups in the United States and United Kingdom. Nat Commun, 13, 636. https://doi.org/10.1038/s41467-022-28200-3

For peer review only

Tables and Figures

Table 1. Sample characteristics (weighted; N=1,408)

Age in years, M (SD)	49.87 (16.14)
Female sex, % (N)	50.4 (709)
White ethnicity, % (N)	89.9 (1261)
Post-16 qualifications, % (N)	68.1 (958)
Health problems, % (N)	44.1 (617)
Perceived high risk of Covid-19, % (N)	11.5 (161)
Being in isolation, % (N)	1.2 (17)
Diagnosed/suspected Covid-19, % (N)	29.7 (418)
Worry about future Covid-19 wave, M (SD)	65.54 (23.36)
Vaccine hesitancy score, M (SD)	1.99 (0.48)
Vaccine knowledge score, M (SD)	7.29 (1.13)
Psychological well-being, M (SD)	3.18 (1.07)

M=Mean; SD=Standard Deviation; Vaccine hesitancy scores range 1-5, with higher score representing higher vaccine hesitancy; Vaccine knowledge scores range 0-9, with higher score representing higher vaccine knowledge; Psychological well-being scores range 1-5, with higher score representing better psychological well-being.

For peer review only

For peer review only - http://bmjopen.bmj.com/site/about/guidelines.xhtml

Table 2. Receipt of/willingness to get vaccinated against Covid-19 for the first time: association with vaccine hesitancy, knowledge, and psychological well-being (unweighted sample).

	**Receipt of/willingness to get an initial vaccine against Covid-19***			
	**N=1,415**		**N=1,406**	
	**OR [95% CI]**	***P***	**aOR [95% CI]**	***P***
Vaccine hesitancy	0.05 [0.03-0.10]	<0.001	0.09 [0.04-0.26]	<0.001
Vaccine knowledge	2.71 [2.17-3.39]	<0.001	1.69 [1.18-2.42]	0.004
Psychological well-being	1.39 [0.97-1.99]	0.072	0.92 [0.56-1.48]	0.719
Post-16 qualifications: yes	1 (ref)		1 (ref)	
No	0.83 [0.28-2.41]	0.728	0.53 [0.19-1.48]	0.227
**Health problems: yes	1 (ref)		1 (ref)	
No	0.55 [0.25-1.21]	0.134	0.80 [0.27-2.43]	0.699
Perceived high risk of Covid-19: no	1 (ref)		1 (ref)	
Yes	1.51 [0.35-6.40]	0.580	1.20 [0.17-8.46]	0.852
Confirmed/suspected Covid-19: no	1 (ref)		1 (ref)	
Yes	0.50 [0.24-1.06]	0.069	1.10 [0.40-3.03]	0.854
Being in isolation: no	1 (ref)		1 (ref)	
Yes	0.20 [0.03-1.64]	0.135	1.29 [0.04-38.77]	0.885
Age (cont.)	1.04 [1.02-1.07]	0.001	1.09 [1.04-1.14]	<0.001
Worry about future Covid-19 wave	1.05 [1.03-1.06]	<0.001	1.03 [1.01-1.05]	<0.001
Time of completion	1.01 [0.96-1.05]	0.843	1.04 [0.97-1.11]	0.308

OR=odds ratio, aOR=adjusted odds ratio, CI=Confidence Interval, ref=reference category.
* Only three participants did not disclose their preference and selected 'don't know'.
**In unadjusted analysis of Health problems N=1,406, as data were missing for some of the participants.

For peer review only - http://bmjopen.bmj.com/site/about/guidelines.xhtml

Table 3. Willingness to get vaccinated against Covid-19 yearly: association with vaccine hesitancy, knowledge, and psychological well-being (unweighted sample).

	**Willingness to get yearly vaccination against Covid-19**			
	**N=1,565**		**N=1,406**	
	**OR [95% CI]**	***p***	**aOR [95% CI]**	***p***
Vaccine hesitancy	0.03 [0.02-0.05]	<0.001	0.05 [0.03-0.09]	<0.001
Vaccine knowledge	2.80 [2.37-3.32]	<0.001	1.81 [1.43-2.29]	<0.001
Psychological well-being	1.39 [1.19-1.62]	<0.001	1.25 [1.02-1.51]	0.014
*Health problems: yes	1 (ref)		1 (ref)	
No	0.99 [0.72-1.36]	0.937	0.98 [0.61-1.54]	0.816
Perceived high risk of Covid-19: no	1 (ref)		1 (ref)	
Yes	1.57 [0.83-2.97]	0.163	2.58 [0.99-6.72]	0.053
Confirmed/suspected Covid-19: no	1 (ref)		1 (ref)	
Yes	0.53 [0.38-0.73]	<0.001	0.77 [0.49-1.21]	0.256
Being in isolation: no	1 (ref)		1 (ref)	
Yes	0.41 [0.11-1.49]	0.173	0.65 [0.07-6.36]	0.714
**Receipt of/willingness to receive an initial Covid-19 vaccine: yes	1 (ref)		1 (ref)	
No	0.01 [0.003-0.04]	<0.001	0.05 [0.01-0.26]	<0.001
Age (cont.)	1.02 [1.01-1.03]	<0.001	1.06 [1.04-1.08]	<0.001
Worry about future Covid-19 wave	1.03 [1.02-1.03]	<0.001	1.03 [1.01-1.05]	0.008
Time of completion	0.98 [0.97-1.00]	0.074	0.98 [0.95-1.01]	0.130

OR=odds ratio, aOR=adjusted odds ratio, CI=Confidence Interval, ref=reference category
*In unadjusted analysis of Health problems N=1,554, as data were missing for some of the participants. ** In unadjusted analysis of Receipt of/willingness to receive Covid-19 vaccine for first time N=1,415, as data were missing for some of the participants.

For peer review only

For peer review only - http://bmjopen.bmj.com/site/about/guidelines.xhtml

Supplementary materials

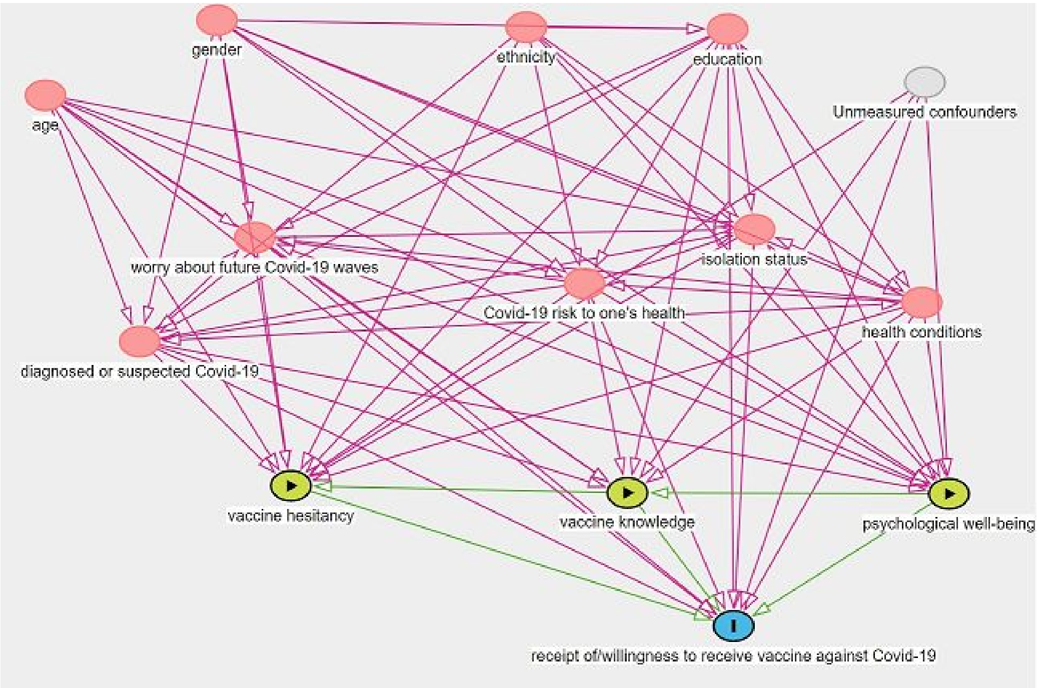

Supplementary figure 1. Directed acyclic graph (DAG) depicting which covariates should be included in a multivariable statistical model in order to minimise bias in the estimate of the total causal effect of i) vaccine hesitancy, ii) vaccine knowledge and iii) psychological well-being during the Covid-19 pandemic and (RQ1i) receipt of/willingness to receive a vaccine against Covid-19. The DAG was developed based on evidence from the existing literature and consensus within the research team.

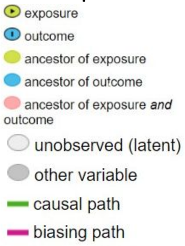

For peer review only

For peer review only - http://bmjopen.bmj.com/site/about/guidelines.xhtml

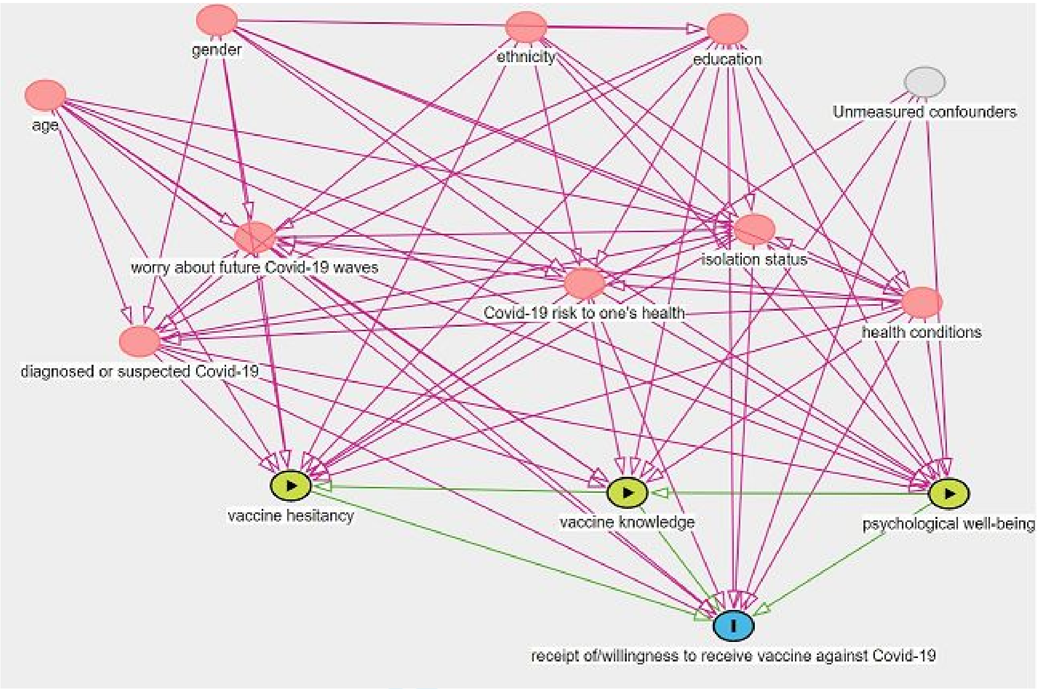

Supplementary figure 2. Directed acyclic graph (DAG) depicting which covariates should be included in a multivariable statistical model in order to minimise bias in the estimate of the total causal effect of i) vaccine hesitancy, ii) vaccine knowledge and iii) psychological well-being during the Covid-19 pandemic and (RQ2) willingness to get vaccinated against Covid-19 on a yearly basis. The DAG was developed based on evidence from the existing literature and consensus within the research team.

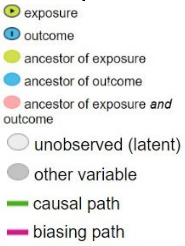

Supplementary table 1. Baseline characteristics of included and excluded sample (unweighted).

	**Total sample N=2,992**	**Included N=1,565 (52.3%)**	**Excluded N=1,427 (47.7%)**	***p***
Age in years M(SD)	47.92 (15.46)	51.74 (14.41)	43.74 (15.49)	<0.001
Female sex, % (N)	68.6 (2,054)	71.3 (1,114)	66.1 (940)	0.002
White ethnicity, % (N)	93.7 (2,804)	95.4 (1,487)	92.6 (1,317)	0.001
Post-16 qualifications, % (N)	86.7 (2,595)	88.9 (1,392)	84.3 (1,203)	<0.001
Health problems, % (N)	41.1 (1,208)	42.4 (659)	39.6 (549)	0.12

M=Mean, SD=Standard Deviation

For peer review only - http://bmjopen.bmj.com/site/about/guidelines.xhtml

Supplementary table 2. Associations between vaccine hesitancy and willingness to get vaccinated against Covid-19 on a yearly basis (sensitivity unweighted analysis).

	**Willingness to get yearly vaccination against Covid-19 (reference category=yes)**							
	**No**				**Not sure**			
	**N=1,565**		**N=1,406**		**N=1,565**		**N=1,406**	
	**OR [95% CI]**	***P***	**aOR [95% CI]**	***P***	**OR [95% CI]**	***P***	**aOR [95% CI]**	***P***
	**N=1,565**							
Vaccine hesitancy	147.70 [67.78-321.86]	<0.001	28.68 [14.38-57.20]	<0.001	17.89 [10.68-29.96]	<0.001	7.94 [4.48-14.08]	<0.001
Vaccine knowledge	0.27 [0.22-0.34]	<0.001	0.47 [0.36-0.62]	<0.001	0.42 [0.34-0.50]	<0.001	0.46 [0.36-0.59]	<0.001
Psychological well-being	0.69 [0.53-0.88]	0.003	1.07 [0.83-1.39]	0.596	0.74 [0.61-0.90]	0.002	0.94 [0.76-1.17]	0.580
*Health problems: yes	1 (ref)		1 (ref)		1 (ref)		1 (ref)	
No	1.30 [0.77-2.22]	0.330	1.35 [0.77-2.36]	0.290	0.85 [0.58-1.26]	0.416	0.82 [0.53-1.27]	0.372
Perceived high risk of Covid-19: no	1 (ref)		1 (ref)		1 (ref)		1 (ref)	
Yes	0.65 [0.23-1.82]	0.411	0.74 [0.28-1.94]	0.545	0.63 [0.29-1.37]	0.247	0.54 [0.24-1.20]	0.128
Confirmed/suspected Covid-19: no	1 (ref)		1 (ref)		1 (ref)		1 (ref)	
Yes	2.50 [1.49-4.17]	<0.001	1.52 [0.89-2.59]	0.122	1.61 [1.08-2.38]	0.022	1.07 [0.68-1.68]	0.773
Being in isolation: no	1 (ref)		1 (ref)		1 (ref)		1 (ref)	
Yes	4.76 [1.00-20.00]	0.049	3.60 [0.39-33.33]	0.261	1.27 [0.16-10.00]	0.821	2.21 [0.10-49.46]	0.618
**Receipt of/willingness to receive an initial Covid-19 vaccine: yes	1 (ref)		1 (ref)		1 (ref)		1 (ref)	
No	-		-		-		-	
Age (cont.)	0.99 [0.97-1.00]	0.087	0.98 [0.96-1.01]	0.123	0.98 [0.97-0.99]	0.002	0.98 [0.96-0.99]	0.005
Worry about future Covid-19 wave	0.96 [0.95-0.97]	<0.001	0.98 [0.97-0.99]	0.001	0.98 [0.97-0.99]	<0.001	1.00 [0.99-1.01]	0.436
Time of completion	1.03 [1.00-1.06]	0.032	1.04 [1.01-1.07]	0.021	1.01 [0.99-1.03]	0.480	1.00 [0.98-1.03]	0.821

OR=odds ratio; aOR=adjusted odds ratio; CI=Confidence Interval; ref=reference category
The reference category for the outcome variable was the response option 'yes'
*In unadjusted analysis of Health problems N=1,554.
** In unadjusted analysis of Receipt of/willingness to receive Covid-19 vaccine for first time N=1,415.

For peer review only

Supplementary materials

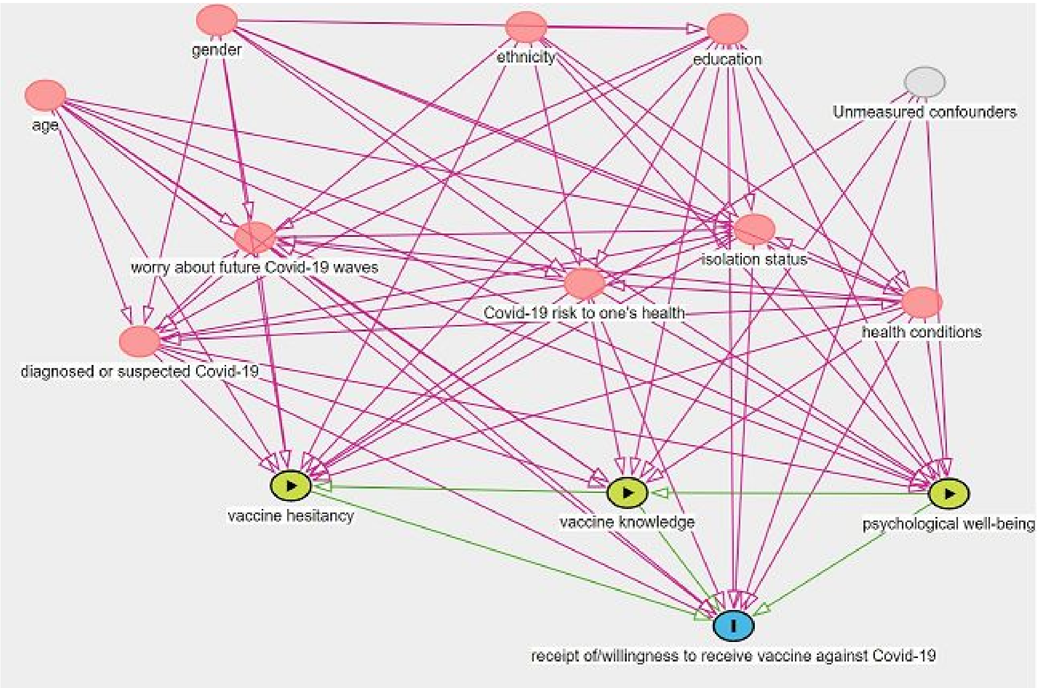

Supplementary figure 1. Directed acyclic graph (DAG) depicting which covariates should be included in a multivariable statistical model in order to minimise bias in the estimate of the total causal effect of i) vaccine hesitancy, ii) vaccine knowledge and iii) psychological well-being during the Covid-19 pandemic and (RQ1i) receipt of/willingness to receive a vaccine against Covid-19. The DAG was developed based on evidence from the existing literature and consensus within the research team.

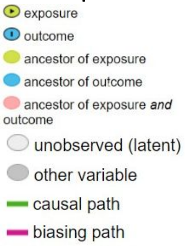

For peer review only

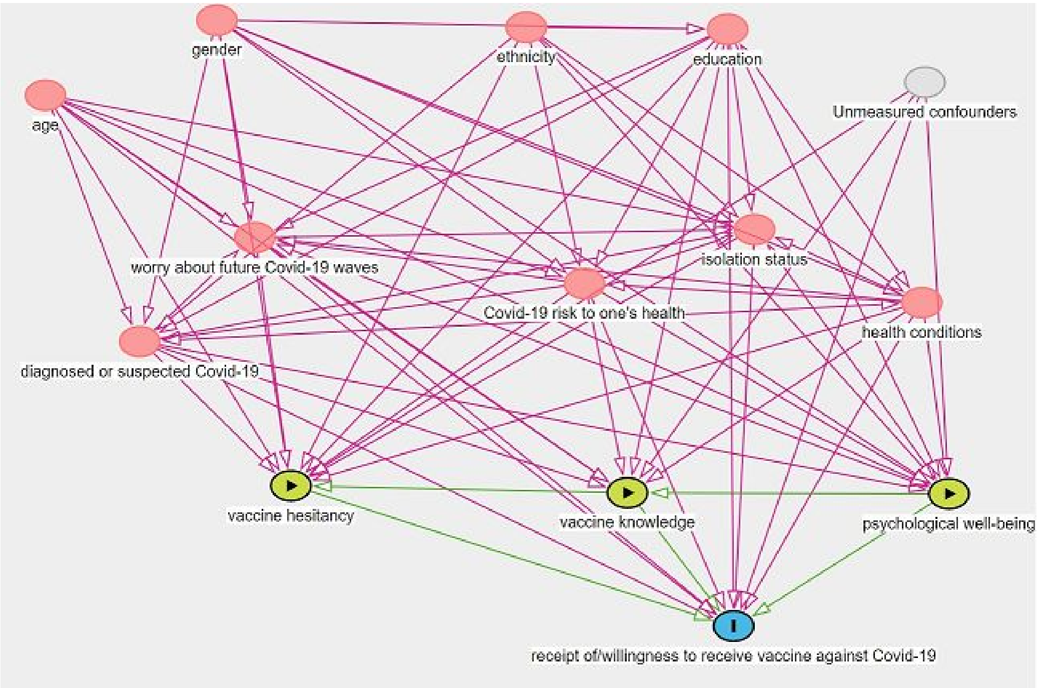

Supplementary figure 2. Directed acyclic graph (DAG) depicting which covariates should be included in a multivariable statistical model in order to minimise bias in the estimate of the total causal effect of i) vaccine hesitancy, ii) vaccine knowledge and iii) psychological well-being during the Covid-19 pandemic and (RQ2) willingness to get vaccinated against Covid-19 on a yearly basis. The DAG was developed based on evidence from the existing literature and consensus within the research team.

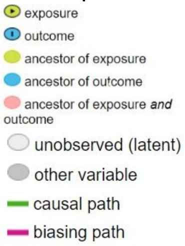

Supplementary table 1. Baseline characteristics of included and excluded sample (unweighted).

	Total sample N=2,992	Included N=1,565 (52.3%)	Excluded N=1,427 (47.7%)	*p*
Age in years M(SD)	47.92 (15.46)	51.74 (14.41)	43.74 (15.49)	<0.001
Female sex, % (N)	68.6 (2,054)	71.3 (1,114)	66.1 (940)	0.002
White ethnicity, % (N)	93.7 (2,804)	95.4 (1,487)	92.6 (1,317)	0.001
Post-16 qualifications, % (N)	86.7 (2,595)	88.9 (1,392)	84.3 (1,203)	<0.001
Health problems, % (N)	41.1 (1,208)	42.4 (659)	39.6 (549)	0.12

M=Mean, SD=Standard Deviation

Supplementary table 2. Associations between vaccine hesitancy and willingness to get vaccinated against Covid-19 on a yearly basis (sensitivity unweighted analysis).

	Willingness to get yearly vaccination against Covid-19 (reference category=yes)							
	No				Not sure			
	N=1,565		N=1,406		N=1,565		N=1,406	
	OR [95% CI]	*P*	aOR [95% CI]	*P*	OR [95% CI]	*P*	aOR [95% CI]	*P*
	N=1,565							
Vaccine hesitancy	147.70 [67.78-321.86]	<0.001	28.68 [14.38-57.20]	<0.001	17.89 [10.68-29.96]	<0.001	7.94 [4.48-14.08]	<0.001
Vaccine knowledge	0.27 [0.22-0.34]	<0.001	0.47 [0.36-0.62]	<0.001	0.42 [0.34-0.50]	<0.001	0.46 [0.36-0.59]	<0.001
Psychological well-being	0.69 [0.53-0.88]	0.003	1.07 [0.83-1.39]	0.596	0.74 [0.61-0.90]	0.002	0.94 [0.76-1.17]	0.580
*Health problems: yes	1 (ref)		1 (ref)		1 (ref)		1 (ref)	
No	1.30 [0.77-2.22]	0.330	1.35 [0.77-2.36]	0.290	0.85 [0.58-1.26]	0.416	0.82 [0.53-1.27]	0.372
Perceived high risk of Covid-19: no	1 (ref)		1 (ref)		1 (ref)		1 (ref)	
Yes	0.65 [0.23-1.82]	0.411	0.74 [0.28-1.94]	0.545	0.63 [0.29-1.37]	0.247	0.54 [0.24-1.20]	0.128
Confirmed/suspected Covid-19: no	1 (ref)		1 (ref)		1 (ref)		1 (ref)	
Yes	2.50 [1.49-4.17]	<0.001	1.52 [0.89-2.59]	0.122	1.61 [1.08-2.38]	0.022	1.07 [0.68-1.68]	0.773
Being in isolation: no	1 (ref)		1 (ref)		1 (ref)		1 (ref)	
Yes	4.76 [1.00-20.00]	0.049	3.60 [0.39-33.33]	0.261	1.27 [0.16-10.00]	0.821	2.21 [0.10-49.46]	0.618
**Receipt of/willingness to receive an initial Covid-19 vaccine: yes	1 (ref)		1 (ref)		1 (ref)		1 (ref)	
No	-		-		-		-	
Age (cont.)	0.99 [0.97-1.00]	0.087	0.98 [0.96-1.01]	0.123	0.98 [0.97-0.99]	0.002	0.98 [0.96-0.99]	0.005
Worry about future Covid-19 wave	0.96 [0.95-0.97]	<0.001	0.98 [0.97-0.99]	0.001	0.98 [0.97-0.99]	<0.001	1.00 [0.99-1.01]	0.436
Time of completion	1.03 [1.00-1.06]	0.032	1.04 [1.01-1.07]	0.021	1.01 [0.99-1.03]	0.480	1.00 [0.98-1.03]	0.821

OR=odds ratio; aOR=adjusted odds ratio; CI=Confidence Interval; ref=reference category
The reference category for the outcome variable was the response option 'yes'
*In unadjusted analysis of Health problems N=1,554.
** In unadjusted analysis of Receipt of/willingness to receive Covid-19 vaccine for first time N=1,415.

STROBE 2007 (v4) Statement—Checklist of items that should be included in reports of *cross-sectional studies*

Section/Topic	Item #	Recommendation	Reported on page #
**Title and abstract**	1	(*a*) Indicate the study's design with a commonly used term in the title or the abstract	1
		(*b*) Provide in the abstract an informative and balanced summary of what was done and what was found	2
**Introduction**			
Background/rationale	2	Explain the scientific background and rationale for the investigation being reported	4-7
Objectives	3	State specific objectives, including any prespecified hypotheses	7-8
**Methods**			
Study design	4	Present key elements of study design early in the paper	8
Setting	5	Describe the setting, locations, and relevant dates, including periods of recruitment, exposure, follow-up, and data collection	8-9
Participants	6	(*a*) Give the eligibility criteria, and the sources and methods of selection of participants	8-9
Variables	7	Clearly define all outcomes, exposures, predictors, potential confounders, and effect modifiers. Give diagnostic criteria, if applicable	9-12
Data sources/ measurement	8*	For each variable of interest, give sources of data and details of methods of assessment (measurement). Describe comparability of assessment methods if there is more than one group	9-12
Bias	9	Describe any efforts to address potential sources of bias	*9-12*
Study size	10	Explain how the study size was arrived at	9-12
Quantitative variables	11	Explain how quantitative variables were handled in the analyses. If applicable, describe which groupings were chosen and why	9-12
Statistical methods	12	(*a*) Describe all statistical methods, including those used to control for confounding	12-14
		(*b*) Describe any methods used to examine subgroups and interactions	12-14
		(*c*) Explain how missing data were addressed	12-14
		(*d*) If applicable, describe analytical methods taking account of sampling strategy	12-14
		(*e*) Describe any sensitivity analyses	10, 13
**Results**			

For peer review only

Participants	13*	(a) Report numbers of individuals at each stage of study—eg numbers potentially eligible, examined for eligibility, confirmed eligible, included in the study, completing follow-up, and analysed	14
		(b) Give reasons for non-participation at each stage	14
		(c) Consider use of a flow diagram	Not applicable
Descriptive data	14*	(a) Give characteristics of study participants (eg demographic, clinical, social) and information on exposures and potential confounders	14, table 1
		(b) Indicate number of participants with missing data for each variable of interest	14
Outcome data	15*	Report numbers of outcome events or summary measures	14-16
Main results	16	(*a*) Give unadjusted estimates and, if applicable, confounder-adjusted estimates and their precision (eg, 95% confidence interval). Make clear which confounders were adjusted for and why they were included	14-16
		(*b*) Report category boundaries when continuous variables were categorized	Not applicable
		(*c*) If relevant, consider translating estimates of relative risk into absolute risk for a meaningful time period	Not applicable
Other analyses	17	Report other analyses done—eg analyses of subgroups and interactions, and sensitivity analyses	14-16
**Discussion**			
Key results	18	Summarise key results with reference to study objectives	16-17
Limitations	19	Discuss limitations of the study, taking into account sources of potential bias or imprecision. Discuss both direction and magnitude of any potential bias	20-21
Interpretation	20	Give a cautious overall interpretation of results considering objectives, limitations, multiplicity of analyses, results from similar studies, and other relevant evidence	16-20
Generalisability	21	Discuss the generalisability (external validity) of the study results	16-20
**Other information**			
Funding	22	Give the source of funding and the role of the funders for the present study and, if applicable, for the original study on which the present article is based	23

*Give information separately for cases and controls in case-control studies and, if applicable, for exposed and unexposed groups in cohort and cross-sectional studies.

Note: An Explanation and Elaboration article discusses each checklist item and gives methodological background and published examples of transparent reporting. The STROBE checklist is best used in conjunction with this article (freely available on the Web sites of PLoS Medicine at http://www.plosmedicine.org/, Annals of Internal Medicine at http://www.annals.org/, and Epidemiology at http://www.epidem.com/). Information on the STROBE Initiative is available at www.strobe-statement.org.

For peer review only

BMJ Open

Willingness to get vaccinated initially and yearly against Covid-19 and its association with vaccine hesitancy, vaccine knowledge and psychological well-being: a cross-sectional study in UK adults

Journal:	*BMJ Open*
Manuscript ID	bmjopen-2023-080778.R1
Article Type:	Original research
Date Submitted by the Author:	22-May-2024

Complete List of Authors:	Kale, Dimitra; University College London, Department of Behavioural Science and Health; University College London Institute of Epidemiology and Health Care<br>Kale, Dimitra; University College London,<br>Shoesmith, Emily; University of York<br>Herbec, Aleksandra; UCL<br>Shahab, Lion; UCL, Dept of Epidemiology and Public Health

**Primary Subject Heading**:	Public health
Secondary Subject Heading:	Mental health
Keywords:	Vaccination, COVID-19, PUBLIC HEALTH, EPIDEMIOLOGY

SCHOLARONE™
Manuscripts

I, the Submitting Author has the right to grant and does grant on behalf of all authors of the Work (as defined in the below author licence), an exclusive licence and/or a non-exclusive licence for contributions from authors who are: i) UK Crown employees; ii) where BMJ has agreed a CC-BY licence shall apply, and/or iii) in accordance with the terms applicable for US Federal Government officers or employees acting as part of their official duties; on a worldwide, perpetual, irrevocable, royalty-free basis to BMJ Publishing Group Ltd ("BMJ") its licensees and where the relevant Journal is co-owned by BMJ to the co-owners of the Journal, to publish the Work in this journal and any other BMJ products and to exploit all rights, as set out in our licence.

The Submitting Author accepts and understands that any supply made under these terms is made by BMJ to the Submitting Author unless you are acting as an employee on behalf of your employer or a postgraduate student of an affiliated institution which is paying any applicable article publishing charge ("APC") for Open Access articles. Where the Submitting Author wishes to make the Work available on an Open Access basis (and intends to pay the relevant APC), the terms of reuse of such Open Access shall be governed by a Creative Commons licence – details of these licences and which Creative Commons licence will apply to this Work are set out in our licence referred to above.

Other than as permitted in any relevant BMJ Author's Self Archiving Policies, I confirm this Work has not been accepted for publication elsewhere, is not being considered for publication elsewhere and does not duplicate material already published. I confirm all authors consent to publication of this Work and authorise the granting of this licence.

For peer review only

Title: Willingness to get vaccinated initially and yearly against Covid-19 and its association with vaccine hesitancy, vaccine knowledge and psychological well-being: a cross-sectional study in UK adults

Authors: Dimitra Kale[1,2], Emily Shoesmith[3], Aleksandra Herbec[1,4], Lion Shahab[1,2]

[1]Department of Behavioural Science and Health, University College London, UK

[2]SPECTRUM Consortium, UK

[3]Department of Health Sciences, University of York, York, United Kingdom

[4]Institute–European Observatory of Health Inequalities, Calisia University, Kalisz, Poland

Corresponding author: Dimitra Kale, dimitra.kale09@ucl.ac.uk

Registration: The analysis plan was pre-registered, and it is available at https://osf.io/s46qm

For peer review only

Abstract

Objectives: This study explores the association between vaccine hesitancy, vaccine knowledge and psychological well-being with (i) receipt of/willingness to receive an initial vaccine against Covid-19, and (ii) willingness to get vaccinated yearly against Covid-19. The importance of different vaccine attributes (e.g., vaccine technology, effectiveness, side effects) to choose a specific Covid-19 vaccine was also assessed.

Design: Cross-sectional survey administered during May-June 2021 on vaccine hesitancy, vaccine knowledge, psychological well-being, willingness to receive Covid-19 vaccines, sociodemographic and Covid-19-related factors.

Setting: United Kingdom.

Participants: A self-selected sample of 1,408 adults.

Outcome measures: Receipt of/willingness to receive Covid-19 vaccine for first time and yearly.

Results: Receipt of/willingness to receive a vaccine against Covid-19 initially and yearly were high (97.0% and 86.6%, respectively). Vaccine hesitancy was negatively associated with receipt of/willingness to receive vaccine initially/yearly (adjusted OR (aOR)=0.09, 95%Confidence Interval (CI) 0.04-0.26, p<0.001/aOR=0.05, 95%CI 0.03-0.09; p<0.001). Vaccine knowledge and psychological well-being were positively associated with willingness to receive a yearly vaccine (aOR=1.81, 95%CI 1.43-2.29, p<0.001 and aOR=1.25, 95%CI 1.02-1.51; p=0.014; respectively) and general vaccine knowledge also with receipt of/willingness to receive vaccine initially (aOR=1.69, 95%CI 1.18-2.42; p=0.004). Vaccine effectiveness was the most important attribute for participants to choose a specific Covid-19 vaccine.

Conclusions: Improving vaccine knowledge and emphasizing vaccine efficacy may minimize vaccine hesitancy and increase Covid-19 vaccine uptake.

For peer review only - http://bmjopen.bmj.com/site/about/guidelines.xhtml

Strength and limitations of this study

- The study benefited from a large sample size.
- The use of validated measures of vaccine hesitancy and vaccine knowledge.
- Examining willingness to get vaccinated against Covid-19 during the roll-out of the first Covid-19 vaccine programme, which improves the validity of findings and minimises recall bias.
- Use of a convenience sample, which may not be representative of the general population.
- Self-reported data and thus susceptible to social desirability bias

Keywords: vaccination, vaccine hesitancy, vaccine, vaccine knowledge, psychological well-being, Covid-19, UK

For peer review only

For peer review only - http://bmjopen.bmj.com/site/about/guidelines.xhtml

Introduction

Covid-19, caused by SARS-CoV-2, is one of the deadliest communicable diseases of the 21st century [1], which led to a pandemic that burdened all aspects of life worldwide [2]. To contain the pandemic, vaccines against Covid-19 were quickly produced and approved for use in the general population in late 2020 and early 2021 across different countries. The development of the Covid-19 vaccines unfolded in real-time, lending a sense that they were developed more rapidly than other vaccines, some used new technology to trigger an immune response (i.e., mRNA technology), raising concerns regarding their safety and effectiveness [3]. Subsequently, these concerns have increased Covid-19 vaccine hesitation, a delay in acceptance or refusal of vaccination despite the availability of vaccination services' [4], around the world [5]. Understanding correlates of Covid-19 vaccine acceptance and vaccine attributes that are important for people when considering getting a Covid-19 vaccine (i.e., vaccine technology) is therefore critical to designing a successful immunization programme that will continue to contain the Covid-19 pandemic and prevent potentially devastating outbreaks in the future. Also, it will help with the development of evidence-based interventions to address vaccine hesitancy in general, which further increases the importance of research in this area.

A growing literature studies the determinants of vaccine hesitancy related to Covid-19 and willingness to get vaccinated against Covid-19. Factors associated with willingness to get vaccinated against Covid-19 include multiple socio-demographics, behavioural and psychological correlates, as well as pandemic-related factors[i.e., 6-16]. Specifically, it has been shown that being older, male, of white ethnicity, with high income, and post-16

For peer review only - http://bmjopen.bmj.com/site/about/guidelines.xhtml

qualification have been consistently related to higher Covid-19 vaccine acceptance and lower Covid-19 vaccine hesitancy [6-10]. Additionally, a positive correlation has been reported between willingness to get vaccinated against Covid-19 and negative affect, with those reporting more worry and anxiety related to Covid-19 also reporting more willingness to get vaccinated against Covid-19 [11, 12]. However, some data suggest that poor psychological well-being and significant levels of depression are associated with vaccine hesitancy [13]. Pandemic experiences, such as history of Covid-19 infection, and higher perceived likelihood of getting Covid-19 [9-11, 15-16], as well as vaccine-related attributes such as efficacy and side effects [17] have also been associated with higher Covid-19 vaccine acceptance] However, most of these studies were assessing hypothetical vaccine acceptance and hesitancy.

Most Covid-19 vaccinations regimes require at least two doses to reach the desired immunization level, and additionally many countries encouraged and facilitated booster doses among its population [18]. Examining rates of Covid-19 vaccination, it seems that they were higher for the first dose of Covid-19 vaccine in comparison with follow-up/booster doses. For example, in the UK the prevalence of receipt of the first Covid-19 vaccination dose was 93.6%, while 88.3% and 70.2% for the second and the third dose, respectively [19]. These rates confirm earlier research, suggesting that a considerable proportion of the general population who have received two doses of a Covid-19 vaccine were either unwilling or unsure about accepting a booster vaccine [12, 13]. Factors associated with this unwillingness and uncertainty include lower levels of stress about catching or becoming seriously ill from Covid-19, lower levels of educational qualification, lower socio-economic position, and younger age [12].

For peer review only - http://bmjopen.bmj.com/site/about/guidelines.xhtml

Emerging data also consistently show that vaccine effectiveness against Covid-19 infection declines over time [20]. Thus, administering additional Covid-19 vaccine doses to appropriate individuals (i.e., elderly, those with vulnerabilities, healthcare professionals) regularly may be necessary to protect susceptible individuals against hospitalisation and death. Indeed, similar to flu vaccines, the Covid-19 vaccine autumn boosters have been offered in UK to vulnerable groups and healthcare professionals [19]. Officials in the US and UK have suggested that we will need to include annual Covid-19 vaccinations, with a schedule similar to flu shots to prevent potentially devastating outbreaks in the future [19, 21]. Crucially, the effectiveness of such programmes depends on vaccine uptake.

While several studies have sought to identify factors associated with willingness to get vaccinated against Covid-19, research examining people's willingness to get vaccinated against Covid-19 on a yearly basis is scarce. For example, Pal et al. [22] examined attitudes towards a hypothetical annual booster vaccine against Covid -19 in US healthcare workers, and they reported that overall acceptance was 83.6% [22]. However, this acceptance was widely divergent among the vaccine-hesitant and non-hesitant group (13.8% versus 89.9%) [22]. Moreover, most studies were conducted prior to the approval of a Covid-19 vaccine [i.e., 9-16]. Thus, it is not known whether these factors have the same importance when the outcome is real rather than hypothetical. Data for the current study were collected during the first Covid-19 vaccine roll-out (May-June 2021), and therefore our participants were asked about their willingness or acceptance of the initial Covid-19 vaccine, and willingness of a yearly Covid-19 vaccination while they had been already offered the first dose of Covid-19 vaccine or were likely to be offered it soon.

For peer review only

For peer review only - http://bmjopen.bmj.com/site/about/guidelines.xhtml

The aim of this study is to explore whether there is an association between vaccine hesitancy, general vaccine knowledge (e.g., the process related to vaccination, the impact of vaccination) and psychological well-being during the Covid-19 pandemic with (i) receipt of/willingness to receive vaccine against Covid-19 for the first time, and (ii) willingness to get vaccinated against Covid-19 on a yearly basis in a sample of UK adults during the period of the first Covid-19 vaccine roll-out. Greater understanding of the associations between vaccine hesitancy, vaccine knowledge, and psychological well-being with willingness to get vaccinated against Covid-19 could help us design mitigating strategies or interventions going forward (i.e., educational, or psychological support). We also assess to what extent different vaccine attributes (i.e., vaccine technology, effectiveness, side effects) are important for people to choose a specific Covid-19 vaccine, if they had the choice, which may also indicate attributes that are important for getting a vaccine against Covid-19.

Research questions:

RQ1: Is there an association between i)vaccine hesitancy, ii)vaccine knowledge and iii)psychological well-being during the Covid-19 pandemic with receipt of/willingness to receive an initial vaccine against Covid-19 in a sample of UK adults during the period of the first Covid-19 vaccine roll-out, adjusting for relevant covariables?

RQ2: Is there an association between i)vaccine hesitancy, ii)vaccine knowledge and iii)psychological well-being during the Covid-19 pandemic with willingness to get vaccinated against Covid-19 on a yearly basis in a sample of UK adults during the period of the first Covid-19 vaccine roll-out, adjusting for relevant covariables?

For peer review only - http://bmjopen.bmj.com/site/about/guidelines.xhtml

RQ3: Which attributes (i.e., vaccine technology, effectiveness, side effects) are most important to people when considering getting a Covid-19 vaccine, if they had the choice?

Methods

Study design

This is a cross-sectional study using data of a longitudinal online survey of adults residing in the UK; the HEalth BEhaviours during the Covid-19 pandemic (HEBECO) study (https://osf.io/sbgru/). The study was approved by the Ethics Committee at the UCL Division of Psychology and Language Sciences (CEHP/2020/759). Baseline data collection occurred between April-June 2020, and follow-up surveys were administered at 1 month, 3, 6 and 12 months from the baseline participation date. The current study uses data from the baseline and 12-month follow-up surveys.

Patient and public involvement statement

In the end of every survey, we asked participants to provide comments related to the content of the surveys, which was considered when the following survey was created. There was no patient and public involvement to the design of the baseline survey, but participants' comments of baseline, 1 month, 3- and 6-month survey were considered when designing the 12-month follow-up survey. Participants and public can access study's results at the study's website, which is freely available.

Study sample

A self-selected sample of UK–based adult (18+ years) who completed both the baseline survey of the HEBECO study between 23/04/2020 (initiation of recruitment) and

For peer review only - http://bmjopen.bmj.com/site/about/guidelines.xhtml

14/06/2020 (inclusive; marking the end of the first national UK lockdown), and a 12-month follow-up survey collected between May-June 2021. At the time of the 12-month follow-up survey, all clinically extremely vulnerable individuals and those aged +40 had been offered at least one dose of Covid-19 vaccine in UK.

Initial recruitment into the baseline survey was online and involved sharing study invitations via multiple channels, including unpaid and paid advertisements on social media (e.g., Facebook), an email campaign across the network of UCL, other universities in UK, Public Health England, Cancer Research UK, charities, and local authorities across the UK. The full recruitment strategy is available online (https://osf.io/sbgru/).

Participants gave their consent prior to data collection. Data were captured and managed by the REDCap electronic data system [23, 24]. Participants were followed-up at 12 months after their baseline survey via email (except for participants that explicitly opted out), with up to three reminders to complete the survey sent at 12-month follow-up.

Measures

The questionnaires were developed by a multi-disciplinary team of academics with input from Cancer Research UK and Public Health England (https://osf.io/sbgru/). The questionnaires included validated items and new items based on expert consensus relating to emerging Covid-19-related aspects, as detailed below. All measures were self-reported.

Outcomes (assessed at 12-month follow-up)

For peer review only - http://bmjopen.bmj.com/site/about/guidelines.xhtml

For peer review only

Receipt of/willingness to receive vaccine against Covid-19 for first time. As Covid-19 vaccines became available in mid-December 2020 in UK, at 12-month follow-up (May-June 2021), individuals reported whether they had been offered a vaccine for Covid-19 (yes/no). Those who replied yes, were asked if they had received a Covid-19 vaccine with the answer option: i)yes, both doses, ii)yes, one dose, iii)no, but I am scheduled to receive the vaccine, iv)no, I decided not to get vaccinated, v)prefer not to say. Participants were classified as willing to receive/received the vaccination if replied (i), (ii) or (iii), and not willing if they selected (iv) and (v). This measure was structured by researchers and was based on previous assessments of Covid-19 vaccine [i.e., 25].

Willingness to get vaccinated against Covid-19 on a yearly basis assessed with a question ‘would you be willing to get vaccinated against the novel coronavirus (SARS-CoV-2) on a yearly basis?’, with the answer options i)yes, ii)no, iii)not sure. Willingness to get vaccinated against Covid-19 on a yearly basis was conceptualised as a binary variable, yes vs no/not sure. We also ran sensitivity analysis where willingness was treated as a 3-category variable yes/no/not sure.

Importance of Covid-19 vaccine attributes was assessed by asking participants to rank in order of importance for them, if they had the choice to select a specific Covid-19 vaccine, the following six attributes; i)vaccine technology (established vs novel), ii)level of protection (how effective it is), iii)side effect profile (how easy it is tolerated), iv)delivery method (injected or not, one dose or more), v)ease of access (walk-in centres vs GP appointment), vi)reputation (manufacturer, media coverage). Participants could rank the attributes from

For peer review only - http://bmjopen.bmj.com/site/about/guidelines.xhtml

1=most important to 6=least important, and they could not assign the same ranking to more than one attribute.

Explanatory variables (assessed at 12-month follow-up):

Vaccine hesitancy was assessed using a nine-item scale [26], revised from the ten-item vaccine hesitancy survey tool developed by the SAGE working group on vaccine hesitancy [27]. The original scale assesses parental attitudes about childhood vaccines, while this adapted version focused on the respondents themselves and is validated in the UK population [27]. Participants were asked to answer nine questions related to their confidence in vaccines in general, rather than vaccines for Covid-19 specifically, on a five-point Likert scale (1=strongly agree to 5=strongly disagree). A continuous score was calculated, with higher scores reflecting a higher level of vaccine hesitancy. Cronbach's alpha values in the present study was 0.70.

Knowledge about vaccines was assessed using a nine-item scale that includes questions about the immunization process related to vaccination, the impact of vaccination and the consequences of vaccination in general, rather than vaccines for Covid-19 specifically. This scale has shown good psychometric properties [28]. Answer options were dichotomised into 1=correct, and 0=incorrect/don't know. A continuous score was calculated, with higher scores presenting a higher knowledge about vaccines. Cronbach's alpha values in the present study was 0.61.

Psychological well-being during the Covid-19 pandemic was assessed with the question 'How would you rate psychological well-being' measured on a scale from 'poor=1' to

For peer review only

'excellent=5'. It was treated as a continuous variable with higher scores presenting better psychological well-being [29].

Covariables

Sociodemographic characteristics were assessed at baseline and included age (continuous in years), gender (female vs all other), education (post-16 qualification vs not), ethnicity (any white ethnicity vs all other/prefer not to say), health conditions (any health condition vs none/prefer not to say).

Covid-19-related characteristics were assessed at 12-month follow-up and include Covid-19 risk to one's health (major risk/significant vs moderate/minor/no risk at all/don't know), isolation status (total vs some/general/no isolation), diagnosed/suspected Covid-19 (yes vs no/prefer not to say), worry about future Covid-19 waves (continuous; 0=not at all, 50=neutral, 100=extremely, higher scores representing more worry).

Confounder variable

Timing (day) of the 12-month follow-up survey completion (daily basis; continuous) was included to control for changes in willingness to get vaccinated in light of the changes in social distancing measures in UK during the period of data collection.

Statistical analysis

Descriptive statistics were calculated to characterise the sample. Data were analysed in SPSS version 27 on complete cases only. The protocol and analysis plan were pre-registered on Open Science Framework (https://osf.io/s46qm). To account for the non-random nature of

For peer review only - http://bmjopen.bmj.com/site/about/guidelines.xhtml

the sampling, data were weighted to the proportions of sex, age, ethnicity, education, and country of living obtained from the Office for National Statistics (2018) for the descriptive analysis. Data were unweighted for the inferential analysis.

Logistic regressions for unweighted data were conducted to examine the association between i)vaccine hesitancy, ii)vaccine knowledge and iii)psychological well-being during the Covid-19 pandemic and (RQ1) receipt of/willingness to receive an initial vaccine against Covid-19, and (RQ2) willingness to get vaccinated against Covid-19 on a yearly basis. We run both unadjusted analysis and adjusted analysis controlling for relevant covariables and timing of 12-month follow-up survey. To identify the relevant covariables for each model, we drew directed acyclic graphs (DAGs) to make the assumptions about the relationships between variables explicit [30]. We used an online tool, DAGitty v3.0 to create the DAGs (http://www.dagitty.net/). Variables and their measurements are depicted by nodes and connected by unidirectional arcs depicting the hypothesised relationship. For RQ1 models, our DAG (supplementary figure 1) implied that the following confounding variables required conditioning (as a minimal sufficient adjustment set): Covid-19 risk to one's health, age, diagnosed or suspected Covid-19, education, health conditions, isolation status, worry about future Covid-19 waves. For RQ2 models, our DAG (supplementary figure 2) implied that the following confounding variables required conditioning (as a minimal sufficient adjustment set): Covid-19 risk to one's health, age, diagnosed or suspected Covid-19, health conditions, isolation status, receipt of/willingness to receive vaccine against Covid-19 for first time, worry about future Covid-19 waves.

For peer review only - http://bmjopen.bmj.com/site/about/guidelines.xhtml

In the sensitivity analysis, multinomial regressions were conducted to examine the association between (i)vaccine hesitancy, (ii)vaccine knowledge and (iii)psychological well-being during the Covid-19 pandemic and willingness to get vaccinated against Covid-19 on a yearly basis, where willingness was treated as a 3-category variable (i)yes, (ii)no, (iii)not sure. The reference category for the outcome variable was the response option 'yes'.

For RQ3, descriptive analysis of the proportions and 95% Confidence Intervals (CI) of the most important vaccine attributes for people to choose a specific Covid-19 vaccine was conducted using weighted data.

For peer review only

Results

Out of a total of 2,992 UK adults recruited into the HEBECO baseline survey, 1,565 (weighted 1,408) participants were successfully followed-up at 12 months and provided data on the variables of interest, and hence met the inclusion criteria for the present analysis. Comparison of those included in the present analysis and those meeting criteria for exclusion showed that included participants were more likely to be older, female, of white ethnicity and have post-16 qualifications (Supplementary Table 1). The weighted characteristics of the included sample are presented in Table 1. Included participants were on average in their fifties, half of them were female, most of them were of white ethnicity and two thirds had post-16 qualifications. Just under half had a health problem, most had low risk perceptions for Covid-19, two thirds were worried about future Covid-19 waves, only 17 were in total isolation, and one third reported diagnosed/suspected Covid-19. The sample had low vaccine hesitancy, high vaccine knowledge and their psychological well-being was average.

For peer review only - http://bmjopen.bmj.com/site/about/guidelines.xhtml

Results RQ1

Of the 1,408 participants included in the present study, 87.2% (95%CI 85.3-89.0) had been offered a vaccine for Covid-19 by the time of the 12-month survey and of these, 97.0% (95%CI 96.0-98.0) had received or they were willing to receive the initial vaccine against Covid-19.

Both unadjusted and adjusted analyses showed a significant negative association between vaccine hesitancy and receipt of/willingness to receive an initial vaccine against Covid-19 (odds ratio (OR)=0.05, 95%CI 0.03-0.10, p<0.001; adjusted OR (aOR)=0.09, 95%CI 0.04-0.26, p<0.001, Table 2). A significant positive association was found between vaccine knowledge and receipt of/willingness to receive a vaccine against Covid-19 for first time (OR=2.71, 95%CI 2.17-3.39, p<0.001; aOR=1.69, 95%CI 1.18-2.42, p=0.004). No significant association was observed between psychological well-being during the Covid-19 pandemic and receipt of/willingness to receive an initial vaccine against Covid-19. Being older and higher levels of worry about future Covid-19 waves were significantly associated with receipt of/willingness to receive an initial vaccine against Covid-19 (Table 2).

Results RQ2

Of the 1,408 participants, 86.6% (95%CI 84.7-88.5) were willing to get vaccinated against Covid-19 on a yearly basis. Both unadjusted and adjusted analyses showed a significant negative association between vaccine hesitancy (OR=0.03, 95%CI 0.02-0.05, p<0.001; aOR=0.05, 95%CI 0.03-0.09; p<0.001) and willingness to get vaccinated against Covid-19 on a yearly basis. Willingness to get yearly vaccinations against Covid-19 was positively

For peer review only - http://bmjopen.bmj.com/site/about/guidelines.xhtml

associated with vaccine knowledge (OR=2.80, 95%CI 2.37-3.32, p<0.001; aOR=1.81, 95%CI 1.43-2.29; p<0.001) and psychological well-being (OR=1.39, 95%CI 1.19-1.62, p<0.001; aOR=1.25, 95%CI 1.02-1.51; p=0.014, Table 3). Being older, higher levels of worry about future Covid-19 waves, and receipt of/willingness to receive an initial Covid-19 vaccine were significantly associated with willingness to get yearly vaccination against Covid-19 (Table 3).

Sensitivity analysis confirmed results, finding a significant negative association between vaccine hesitancy and willingness to get vaccinated against Covid-19 on a yearly basis (Supplementary Table 2). Similarly, there was a significant positive association between vaccine knowledge and psychological well-being with willingness to get yearly vaccines against Covid-19 (Supplementary Table 2).

Results RQ3

By far the most important vaccine attribute for participants to choose a specific Covid-19 vaccine was effectiveness (57.2%, 95%CI 54.5-59.8), while the least important was related to reputation of the vaccine based on the manufacturer and media coverage (9.7%, 95%CI 8.2-11.3). All other vaccine attributes were rated as similarly unimportant; vaccine technology: 13.3%, 95%CI 11.6-15.2; side-effects: 13.6%, 95%CI 11.9-15.5; delivery method: 12.4%, 95%CI 10.7-14.2; and ease of access: 13.4% 95%CI 11.6-15.2.

Discussion

In this sample of UK adults, vaccine hesitancy was negatively associated with receipt of/willingness to receive a first vaccine against Covid-19 and willingness to get a yearly Covid-19 vaccine. Vaccine knowledge was positively associated with receipt of/willingness

For peer review only - http://bmjopen.bmj.com/site/about/guidelines.xhtml

to receive the initial vaccine against Covid-19 and willingness to get a yearly Covid-19 vaccine, while there was also a positive association between psychological well-being during the Covid-19 pandemic and willingness to get a yearly Covid-19 vaccine only. Effectiveness of the vaccine was the most important attribute for participants to choose a specific Covid-19 vaccine if they had the choice, while the least important was reputation of the vaccine based on the manufacturer and media coverage.

It should be noted that in the current study an overwhelming majority of respondents had either received or were willing to receive the first dose of Covid-19 vaccine, while vaccine hesitancy was low. Previous studies conducted in the UK have documented higher vaccine hesitancy and lower vaccine acceptance rates (between 63% - 69%; e.g. [31, 32]). However, most previous work was carried out in the early days of the Covid-19 vaccine roll out, while our data were collected more than half-way through the vaccination programme in the UK. At that point of data collection, people might have had more confidence in the safety and effectiveness of Covid-19 vaccine, since not many side effects were reported from individuals who had already received the vaccine. It was also a period when Covid-19 measures were favourable for people who were vaccinated (i.e., fully vaccinated did not have to quarantine when travelling back to England), which might have also influenced individuals' decision to get vaccinated in order to enable them to resume travelling and other activities and return to 'normal living' after a long period of social distancing measures and quarantines. Longitudinal and cross-sectional studies also have found that Covid-19 vaccination intention has increased over time in the UK [7, 33], with high compliance rates [19].

For peer review only - http://bmjopen.bmj.com/site/about/guidelines.xhtml

The present study's high percentage of Covid-19 vaccine acceptance might also be attributed to the sociodemographic characteristics of the participants. For example, vaccine acceptance has been linked with being older, male, of white ethnicity, and having higher qualification [i.e., 6-8], which reflects our sample characteristics. However, it should be noted that we weighted the sample to be representative of the population, so this cannot entirely explain the high acceptance rates observed.

Willingness to get vaccinated against Covid-19 on a yearly basis was also high, but lower than for the first dose of Covid-19 vaccine, and similar to previous research on US healthcare workers, which suggested an acceptance rate of a hypothetical annual booster vaccine against Covid -19 of 83.6% [22]. The lower levels of willingness to get a yearly Covid-19 vaccine compared with the first dose might be attributed to the fact that participants were less worried about a future wave of Covid-19, thus assuming they will not need protection against Covid-19 provided by the vaccine. Additionally, since people know that boosters are reserved for special populations (i.e., all adults aged 50 years and over, healthcare professionals, and those with a clinical risk [19], they may think yearly vaccinations are unnecessary for themselves.

Vaccine knowledge was high in our sample, and it was positively associated with willingness to get vaccinated against Covid-19 initially and on a yearly basis. This may suggest that individuals who are better informed about vaccines in general have a greater understanding of the risk-benefit calculus of vaccinations, and thus of the benefits of the Covid-19 vaccine, specifically, for individual and population health. Additionally, most of the participants had post-16 qualifications, and it has been documented that participant with a bachelor degree

For peer review only - http://bmjopen.bmj.com/site/about/guidelines.xhtml

are more likely to have a higher health literacy leading to a greater understanding of vaccine development and clinical trials to stop the spread of disease [34]. Earlier research also suggests that insufficient knowledge about the new Covid-19 vaccine and fears of long-term side effects were reasons not to get vaccinated. However, as more knowledge about the vaccine accumulated, and people become more educated about the vaccines, this may have increases willingness to accept the Covid-19 vaccine [35-37].

Psychological well-being in our sample was average and positively associated only with willingness to get a yearly Covid-19 vaccine, and not receipt of/willingness to receive the initial Covid-19 vaccine. The average levels of psychological well-being reported by our sample may be due to the timing of data collection. High levels of psychological distress were documented during nationwide lockdowns and when infection levels and subsequent death rates were at their peak [i.e., 38]. Though our study was conducted at a time when a new phase in the UK government's response to the pandemic was underway, moving away from stringent restrictions on day-to-day lives, towards advising people on how to protect themselves and others [39] and allowing for more socialisation. Additionally, an effective vaccine had already been developed and deployed. Previous research has found conflicting results about the association between psychological well-being and receipt of Covid-19 vaccine, with some findings linking poor psychological well-being, high levels of anxiety and worry with willingness to get vaccinated against Covid-19 [11, 12], and others reporting significant association between poor psychological well-being and high levels of vaccine hesitancy [13]. Our results also suggest that people with higher levels of psychological well-being were more willing to get vaccinated against Covid-19 on a yearly basis. It could be argued that people with better psychological well-being may be less prone to worry/be

For peer review only - http://bmjopen.bmj.com/site/about/guidelines.xhtml

anxious when it comes to trying new products/vaccine, and thus more willing to get vaccinated against Covid-19.

Consistent with previous studies [i.e., 33, 40] our findings indicate that vaccine efficacy is one of the most important determinants for Covid-19 vaccine acceptance. From a practical perspective, promotional materials could emphasize Covid-19 vaccine efficacy as a strategy to enhance the willingness for Covid-19 vaccine uptake. Aside from dispelling misinformation about vaccines, it may also be helpful to provide individuals with more information on why a particular type of vaccine can be effective.

Strengths and Limitations

The study benefited from a large sample size, use of validated measures of vaccine hesitancy and vaccine knowledge, as well as from examining willingness to get vaccinated against Covid-19 during a declared pandemic, and at a time during the roll-out of the first Covid-19 vaccine programme. This improves the validity of findings and minimises recall bias as the study was conducted when vaccination uptake was being actively promoted by government and health promotion authorities, and participants were faced with the actual question on whether or not to get vaccinated.

The study also has several limitations, including the use of a convenience sample who may have participated in the study due to a higher interest in the pandemic than in the general population. This interest may also be related to a higher receipt/willingness to get vaccinated against Covid-19. Additionally, the majority of participants were of white ethnicity, which meant we could not look at other ethnicities separately, when ethnic

For peer review only - http://bmjopen.bmj.com/site/about/guidelines.xhtml

disparities in vaccine uptake/hesitancy have been reported elsewhere [41]. All data were self-reported and thus susceptible to social desirability bias. However, our rates of receipt/willingness to receive the first dose of Covid-19 vaccine were similar to the UK government rates of the first dose of Covid-19 vaccine [19]. Additionally, receipt of/willingness to receive an initial vaccine against Covid-19 was assessed only among those who have been offered a vaccine against Covid-19 at the time of the 12-month survey, thus excluding some younger participants.

Conclusions

In conclusion, our findings suggest that willingness to get vaccinated against Covid-19 in a sample of UK adults during the time of the vaccine roll out was high, vaccine hesitancy was low, general vaccine knowledge was high and psychological well-being was average. There was a negative association between vaccine hesitancy and acceptance/willingness to receive the initial dose of a Covid-19 vaccine and a yearly booster, a positive association between vaccine knowledge and willingness to receive both initial and yearly doses of a Covid-19 vaccine, while there was also a positive association between psychological well-being during the Covid-19 pandemic and willingness to get a yearly Covid-19 vaccine. Effectiveness of the vaccine was the most important attribute for participants to choose a specific Covid-19 vaccine, if they had the choice. Our findings suggest that improving knowledge about vaccines and emphasizing vaccine efficacy could minimise vaccine hesitancy and enhance the willingness for Covid-19 vaccine uptake. Thus, tailored education on vaccine hesitancy along with communication strategies that highlight vaccine efficacy are crucial to improve vaccination rates during the Covid-19 pandemic and future outbreaks.

For peer review only - http://bmjopen.bmj.com/site/about/guidelines.xhtml

(4500 words)

Declarations

Ethics approval and consent to participate

The study has been approved by UCL Research Ethics Committee at the UCL Division of Psychology and Language Sciences (PaLS) (CEHP/2020/579) as part of the larger programme ‘The optimisation and implementation of interventions to change behaviours related to health and the environment’. The authors assert that all procedures contributing to this work comply with the ethical standards of the relevant national and institutional committees on human experimentation and with the Helsinki Declaration of 1975, as revised in 2008. All participants provided fully informed consent. The study is GDPR compliant.

Consent for publication

Not applicable.

Availability of data

The dataset used and analysed during the current study are available from the corresponding author (Dimitra Kale) on reasonable request.

For peer review only - http://bmjopen.bmj.com/site/about/guidelines.xhtml

Competing Interests

DK, ES, AH declare no conflicts of interest. LS has received honoraria for talks, an unrestricted research grant and travel expenses to attend meetings and workshops from Pfizer and has acted as paid reviewer for grant awarding bodies and as a paid consultant for health care companies.

Funding

This project is partially funded by an ongoing Cancer Research UK (CRUK) Programme Grant to UCL Tobacco and Alcohol Research Group [PRCRPG-Nov21\100002] and by SPECTRUM a UK Prevention Research Partnership Consortium [grant number MR/S037519/1]. Funders had no role in the design and conduct of the study; collection, management, analysis and interpretation of the data; preparation, review or approval of the manuscript; and decision to submit the manuscript for publication. For the purpose of Open Access, the author has applied a CC BY public copyright licence to any Author Accepted Manuscript version arising from this submission.

Author contribution

LS, AH, and DK conceived and designed the study. DK analysed the data and wrote the first draft. LS, ES and AH provided critical revisions. All authors read and approved the submitted manuscript. DK serves as guarantor and accepts full responsibility for the work and/or the conduct of the study, had access to the data, and controlled the decision to publish.

Acknowledgements

For peer review only

We are grateful to all participants who have been supporting our research. We would like to thank Public Health England, and particularly members of the Behavioural Insights at Public Health England for providing feedback on the survey wording. We would like to thank Public Health England, Cancer Research UK, local authorities, Mayors' offices, as well as charities and other organisations in the UK, including the Asthma UK and British Lung Foundation Partnership, for supporting our recruitment campaign.

References

[1] Wilder-Smith, A. 2021. COVID-19 in comparison with other emerging viral diseases: risk of geographic spread via travel. Trop Dis Travel Med Vaccines 7, 3.

[2] Keni, R., Alexander, A., Nayak, P. G., Mudgal, J., Nandakumar, K., 2020. COVID-19: Emergence, spread, possible treatments, and global burden. Frontiers in Public Health, 8. https://doi.org/10.3389/fpubh.2020.00216

[3] Chavda, V.P., Chen, Y., Dave, J., Chen, Z., Chauhan, S.C., Yallapu, M.M., Uversky, V.N., Bezbaruah, R., Patel, S., Apostolopoulos V., 2022. COVID-19 and vaccination: myths vs science, Expert Review of Vaccines, 21:11, 1603-1620, doi: 10.1080/14760584.2022.2114900

[4] MacDonald, N. E.; SAGE Working Group on Vaccine Hesitancy, 2015. Vaccine hesitancy: definition, scope and determinants. Vaccine, 33, 4161–4164

[5] Sallam, M., 2021. COVID-19 Vaccine Hesitancy Worldwide: A Concise Systematic Review of Vaccine Acceptance Rates. Vaccines, 16;9(2):160. doi: 10.3390/vaccines9020160. PMID: 33669441; PMCID: PMC7920465.

For peer review only

For peer review only - http://bmjopen.bmj.com/site/about/guidelines.xhtml

[6] Kafadar, A.H., Tekeli, G.G., Jones, K.A., Blossom S., Dening, T., 2023. Determinants for COVID-19 vaccine hesitancy in the general population: a systematic review of reviews. J Public Health (Berl.) 31, 1829–1845.

[7] Paul, E., Steptoe, A., Fancourt, D., 2021. Attitudes towards vaccines and intention to vaccinate against COVID-19: implications for public health communications. Lancet Reg Health - Eur. 1(100012)

[8] Fisher, K.A., Bloomstone, S.J., Walder, J., Crawford, S., Fouayzi, H., Mazor, K.M., 2020. Attitudes toward a potential SARS-CoV-2 vaccine. Ann. Intern. Med. 173, 964–973

[9] Yang, J., Liao, Y., Hua, Q., LV, H. 2022. A survey of awareness of Covid-19 knowledge, willingness and influencing factors of Covid-19 vaccination. Vaccines. 10(4):524

[10] Reiter, P.L., Pennell, M.L., Katz, M.L., 2020. Acceptability of a COVID-19 vaccine among adults in the United States: how many people would get vaccinated? Vaccine 38, 6500–6507. https://doi.org/10.1016/j.vaccine.2020.08.043.

[11] Kejriwal, M., Shen, K., 2021. COVID-19 vaccine hesitancy is positively associated with affective wellbeing. https://doi.org/10.31234/osf.io/nkvhs

[12] Paul, E., Fancourt, D., 2022. Predictors of uncertainty and unwillingness to receive the COVID-19 booster vaccine: An observational study of 22,139 fully vaccinated adults in the UK. The Lancet Regional Health – Europe, Volume 14, 100317

[13] Galanis, P., Vraka, I., Katsiroumpa, A., Siskou, O., Konstantakopoulou, O., Katsoulas, T., Mariolis-Sapsakos, T., Kaitelidou, D., 2022. First COVID-19 Booster Dose in the General Population: A Systematic Review and Meta-Analysis of Willingness and Its Predictors. Vaccine., 8;10(7):1097. doi: 10.3390/vaccines10071097.

For peer review only

[14] Kim, H.L., Eun Kim, E., Han, D.H., 2023. Effect of psychological factors on COVID-19 vaccine hesitancy, Journal of Public Health, 45(3): e522–e531, https://doi.org/10.1093/pubmed/fdad050 .

[15] Khubchandani, J., Sharma, S., Price, J.H., Wiblishauser, M.J., Sharma, M., Webb, F.J., 2021. COVID-19 vaccination hesitancy in the United States: a rapid national assessment. J. Community Health 46, 270–277. https://doi.org/10.1007/s10900-020-00958-x.

[16] Killgore, W.D.S., Cloonan, S.A., Taylor, E.C., Dailey, N.S., 2021. The COVID-19 vaccine is here—now who is willing to get it? Vaccines 9, 339. https://doi.org/10.3390/ vaccines9040339.

[17] Kreps, S., Prasad, S., Brownstein, J.S., Hswen, Y., Garibaldi, B.T., Zhang, B., Kriner, D. L., 2020. Factors Associated With US Adults' Likelihood of Accepting COVID-19 Vaccination. JAMA Netw Open. 3(10):e2025594. doi:10.1001/jamanetworkopen.2020.25594

[18] World Health Organization (WHO), 2022. COVID-19 advice for the public: Getting vaccinated. https://www.who.int/emergencies/diseases/novel-coronavirus-2019/covid-19-vaccines/advice#:~:text=Getting%20a%20third%20dose%20can,when%20it%20is%20your%20turn.&text=It%20is%20safe%20for%20most,get%20vaccinated%20against%20COVID%2D19. Accessed December 2022.

[19] GOV.UK, 2022. Vaccinations in United Kingdom. https://coronavirus.data.gov.uk/details/vaccinations. Accessed November 2022

For peer review only

For peer review only - http://bmjopen.bmj.com/site/about/guidelines.xhtml

[20] Ferdinands, J.M., Rao, S., Dixon, B.E., Mitchell, P.K., DeSilva, M.B., Irving, S.A. et al., 2022. Waning of vaccine effectiveness against moderate and severe covid-19 among adults in the US from the VISION network: test negative, case-control study BMJ, 379 :e072141, doi:10.1136/bmj-2022-072141

[21] Public Health Agency, 2022. COVID-19 vaccination - A guide to the autumn booster. https://www.publichealth.hscni.net/publications/covid-19-vaccination-guide-autumn-booster. Accessed November 2022.

[22] Pal, S., Shekhar, R., Kottewar, S., Upadhyay, S., Singh, M., Pathak, D., Kapuria, D., Barrett, E., Sheikh, A.B., 2021. COVID-19 Vaccine Hesitancy and Attitude toward Booster Doses among US Healthcare Workers. Vaccines 2021, 9, 1358. https://doi.org/10.3390/vaccines9111358

[23] Harris, P.A., Taylor, R., Thielke, R., Payne, J., Gonzalez, N., Conde, J.G., 2009. Research electronic data capture (REDCap)—A metadata driven methodology and workflow process for providing translational research informatics support. J. Biomed. Inform. 42, 377–381.

[24] Harris, P.A., Taylor, R., Minor, B.L., Elliott, V., Fernandez, M., O'Neal, L., McLeod, L., Delacqua, G., Delacqua, F., Kirby, J., 2019. The REDCap consortium: Building an international community of software platform partners. J. Biomed. Inform. 95, 103208.

[25] Household Pulse Survey, 2021. Measuring household experiences during the coronavirus pandemic. Census.gov. https://www.census.gov/data/experimental-data-products/household-pulse-survey.html

For peer review only - http://bmjopen.bmj.com/site/about/guidelines.xhtml

[26] Luyten, J., Bruyneel, L., van Hoek, A.J., 2019. Assessing vaccine hesitancy in the UK population using a generalized vaccine hesitancy survey instrument. Vaccine. 24;37(18):2494-2501. doi: 10.1016/j.vaccine.2019.03.041

[27] Larson, H. J., Jarrett, C., Schulz, W. S., Chaudhuri, M., Zhou,Y., Dube, E. et al.; SAGE Working Group on Vaccine Hesitancy, 2015. Measuring vaccine hesitancy: the development of a survey tool. Vaccine, 33, 4165–4175

[28] Zingg, A., Siegrist, M., 2012. Measuring people's knowledge about vaccination: Developing a one-dimensional scale. Vaccine. 30:3771-3777. https://doi.org/10.1016/j.vaccine.2012.03.014

[29] Dicken, S.J., Mitchell, J.J., Newberry Le Vay, J., Beard, E., Kale, D., Herbec, A., Shahab, L., 2021. Impact of COVID-19 pandemic on weight and BMI among UK adults: a longitudinal analysis of data from the HEBECO study. Nutrients. 2021; 13(9):2911, doi:10.3390/nu13092911

[30] Tennant, P.W.G., Murray, E.J., Arnold, K.F., Berrie, L., Fox, M.P., Gadd, S.C., et al., 2021. Use of directed acyclic graphs (DAGs) to identify confounders in applied health research: review and recommendations. Int J Epidemiol. 50(2):620–32.

[31] Murphy, J., Vallières, F., Bentall, R.P., Shevlin, M., McBride, O., Hartman, T.K., McKay, R., Bennett, K., Mason, L., Gibson-Miller, J., Levita, L., Martinez, A.P., Stocks, T.V.A., Karatzias, T., Hyland, P., 2021.. Psychological characteristics associated with COVID-19 vaccine hesitancy and resistance in Ireland and the United Kingdom. Nat Commun 12, 29 (2021). https://doi.org/10.1038/s41467-020-20226-9

For peer review only

For peer review only - http://bmjopen.bmj.com/site/about/guidelines.xhtml

[32] Sherman, S.M., Smith, L.E., Sim, J., Amlôt, R., Cutts, M., Dasch, H., Rubin, G.J., Sevdalis, N., 2021. COVID-19 vaccination intention in the UK: results from the COVID-19 vaccination acceptability study (CoVAccS), a nationally representative cross-sectional survey. Hum Vaccin Immunother. 3;17(6):1612-1621. doi: 10.1080/21645515.2020.1846397.

[33] Byrne, T., Patel, P., Shrotri, M., Beale, S., Michie, S., Butt, J., Hawkins, N., Hardelid, P., Rodger, A., Aryee, A., Braithwaite, I., Fong, W.L.E., Fragaszy, E., Geismar, C., Kovar, J., Navaratnam, A.M.D., Nguyen, V., Hayward, A., Aldridge. R.W.; Virus Watch Collaborative. 2021. Trends, patterns and psychological influences on COVID-19 vaccination intention: Findings from a large prospective community cohort study in England and Wales (Virus Watch). Vaccine. 26;39(48):7108-7116. doi: 10.1016/j.vaccine.2021.09.066.

[34] Sukys, S., Cesnaitiene, V.J., Ossowsky, Z.M. 2017. Is health education at university associated with students' health literacy? Evidence from cross-sectional study applying HLS-EU-Q. Biomed. Res. Int. 8516843

[35] Grech, V., Gauci, C., Agius, S. 2020. Withdrawn: Vaccine hesitancy among Maltese Healthcare workers toward influenza and novel COVID-19 vaccination. Early Hum Dev. 1:105213. doi: 10.1016/j.earlhumdev.2020.105213.

[36] Kwok, K.O., Li, K.K., Wei, W.I., Tang, A., Wong, S.Y.S., Lee, S.S. 2021. Influenza vaccine uptake, COVID-19 vaccination intention and vaccine hesitancy among nurses: A survey. International Journal of Nursing Studies, 114

For peer review only

For peer review only - http://bmjopen.bmj.com/site/about/guidelines.xhtml

[37] Rabi, R., Maraqa, B., Nazzal, Z., Zink, T. 2021. Factors affecting nurses' intention to accept the COVID-19 vaccine: A cross-sectional study. Public Health Nurs. 38(5):781-788. doi: 10.1111/phn.12907

[38] Aknin, L. B., Andretti, B., Goldszmidt, R., Helliwell, J. F., Petherick, A., De Neve, J., Dunn, E. W., Fancourt, D., Goldberg, E., Jones, S. P., Karadag, O., Karam, E., Layard, R., Saxena, S., Thornton, E., Whillans, A., Zaki, J. 2022. Policy stringency and mental health during the COVID-19 pandemic: a longitudinal analysis of data from 15 countries, The Lancet Public Health, 7(5): e417-e426

[39] GOV.UK, 2021. Guidance. COVID-19 response: Summer 2021. https://www.gov.uk/government/publications/covid-19-response-summer-2021-roadmap/covid-19-response-summer-2021. Acessed March 2023

[40] Freeman, D., Loe, B.S., Chadwick, A., Vaccari, C., Waite, F., Rosebrock, L., Jenner, L., Petit, A., Lewandowsky, S., Vanderslott, S., Innocenti, S., Larkin, M., Giubilini, A., Yu, L.M., McShane, H., Pollard, A.J., Lambe, S. 2022. COVID-19 vaccine hesitancy in the UK: the Oxford coronavirus explanations, attitudes, and narratives survey (Oceans) II. Psychol Med. 52(14):3127-3141. doi: 10.1017/S0033291720005188

[41] Nguyen, L.H., Joshi, A.D., Drew, D.A. et al. (2022). Self-reported COVID-19 vaccine hesitancy and uptake among participants from different racial and ethnic groups in the United States and United Kingdom. Nat Commun, 13, 636. https://doi.org/10.1038/s41467-022-28200-3

For peer review only - http://bmjopen.bmj.com/site/about/guidelines.xhtml

692 **Tables and Figures**

693

694 Table 1. Sample characteristics (weighted; N=1,408)

Age in years, M (SD)	49.87 (16.14)
Female sex, % (N)	50.4 (709)
White ethnicity, % (N)	89.9 (1261)
Post-16 qualifications, % (N)	68.1 (958)
Health problems, % (N)	44.1 (617)
Perceived high risk of Covid-19, % (N)	11.5 (161)
Being in isolation, % (N)	1.2 (17)
Diagnosed/suspected Covid-19, % (N)	29.7 (418)
Worry about future Covid-19 wave, M (SD)	65.54 (23.36)
Vaccine hesitancy score, M (SD)	1.99 (0.48)
Vaccine knowledge score, M (SD)	7.29 (1.13)
Psychological well-being, M (SD)	3.18 (1.07)

695 M=Mean; SD=Standard Deviation; Vaccine hesitancy scores range 1-5, with higher score
696 representing higher vaccine hesitancy; Vaccine knowledge scores range 0-9, with higher score
697 representing higher vaccine knowledge; Psychological well-being scores range 1-5, with higher score
698 representing better psychological well-being.

699

700

For peer review only

Table 2. Receipt of/willingness to get vaccinated against Covid-19 for the first time: association with vaccine hesitancy, knowledge, and psychological well-being (unweighted sample).

	**Receipt of/willingness to get an initial vaccine against Covid-19***			
	**N=1,415**		**N=1,406**	
	**OR [95% CI]**	***P***	**aOR [95% CI]**	***P***
Vaccine hesitancy	0.05 [0.03-0.10]	<0.001	0.09 [0.04-0.26]	<0.001
Vaccine knowledge	2.71 [2.17-3.39]	<0.001	1.69 [1.18-2.42]	0.004
Psychological well-being	1.39 [0.97-1.99]	0.072	0.92 [0.56-1.48]	0.719
Post-16 qualifications: yes	1 (ref)		1 (ref)	
No	0.83 [0.28-2.41]	0.728	0.53 [0.19-1.48]	0.227
**Health problems: yes	1 (ref)		1 (ref)	
No	0.55 [0.25-1.21]	0.134	0.80 [0.27-2.43]	0.699
Perceived high risk of Covid-19: no	1 (ref)		1 (ref)	
Yes	1.51 [0.35-6.40]	0.580	1.20 [0.17-8.46]	0.852
Confirmed/suspected Covid-19: no	1 (ref)		1 (ref)	
Yes	0.50 [0.24-1.06]	0.069	1.10 [0.40-3.03]	0.854
Being in isolation: no	1 (ref)		1 (ref)	
Yes	0.20 [0.03-1.64]	0.135	1.29 [0.04-38.77]	0.885
Age (cont.)	1.04 [1.02-1.07]	0.001	1.09 [1.04-1.14]	<0.001
Worry about future Covid-19 wave	1.05 [1.03-1.06]	<0.001	1.03 [1.01-1.05]	<0.001
Time of completion	1.01 [0.96-1.05]	0.843	1.04 [0.97-1.11]	0.308

OR=odds ratio, aOR=adjusted odds ratio, CI=Confidence Interval, ref=reference category.
* Only three participants did not disclose their preference and selected 'don't know'.
**In unadjusted analysis of Health problems N=1,406, as data were missing for some of the participants.

For peer review only

For peer review only - http://bmjopen.bmj.com/site/about/guidelines.xhtml

709 Table 3. Willingness to get vaccinated against Covid-19 yearly: association with vaccine hesitancy,
710 knowledge, and psychological well-being (unweighted sample).

	**Willingness to get yearly vaccination against Covid-19**			
	**N=1,565**		**N=1,406**	
	**OR [95% CI]**	***p***	**aOR [95% CI]**	***p***
Vaccine hesitancy	0.03 [0.02-0.05]	<0.001	0.05 [0.03-0.09]	<0.001
Vaccine knowledge	2.80 [2.37-3.32]	<0.001	1.81 [1.43-2.29]	<0.001
Psychological well-being	1.39 [1.19-1.62]	<0.001	1.25 [1.02-1.51]	0.014
*Health problems: yes	1 (ref)		1 (ref)	
No	0.99 [0.72-1.36]	0.937	0.98 [0.61-1.54]	0.816
Perceived high risk of Covid-19: no	1 (ref)		1 (ref)	
Yes	1.57 [0.83-2.97]	0.163	2.58 [0.99-6.72]	0.053
Confirmed/suspected Covid-19: no	1 (ref)		1 (ref)	
Yes	0.53 [0.38-0.73]	<0.001	0.77 [0.49-1.21]	0.256
Being in isolation: no	1 (ref)		1 (ref)	
Yes	0.41 [0.11-1.49]	0.173	0.65 [0.07-6.36]	0.714
**Receipt of/willingness to receive an initial Covid-19 vaccine: yes	1 (ref)		1 (ref)	
No	0.01 [0.003-0.04]	<0.001	0.05 [0.01-0.26]	<0.001
Age (cont.)	1.02 [1.01-1.03]	<0.001	1.06 [1.04-1.08]	<0.001
Worry about future Covid-19 wave	1.03 [1.02-1.03]	<0.001	1.03 [1.01-1.05]	0.008
Time of completion	0.98 [0.97-1.00]	0.074	0.98 [0.95-1.01]	0.130

711 OR=odds ratio, aOR=adjusted odds ratio, CI=Confidence Interval, ref=reference category
712 *In unadjusted analysis of Health problems N=1,554, as data were missing for some of the
713 participants. ** In unadjusted analysis of Receipt of/willingness to receive Covid-19 vaccine for first
714 time N=1,415, as data were missing for some of the participants.

For peer review only

Supplementary materials

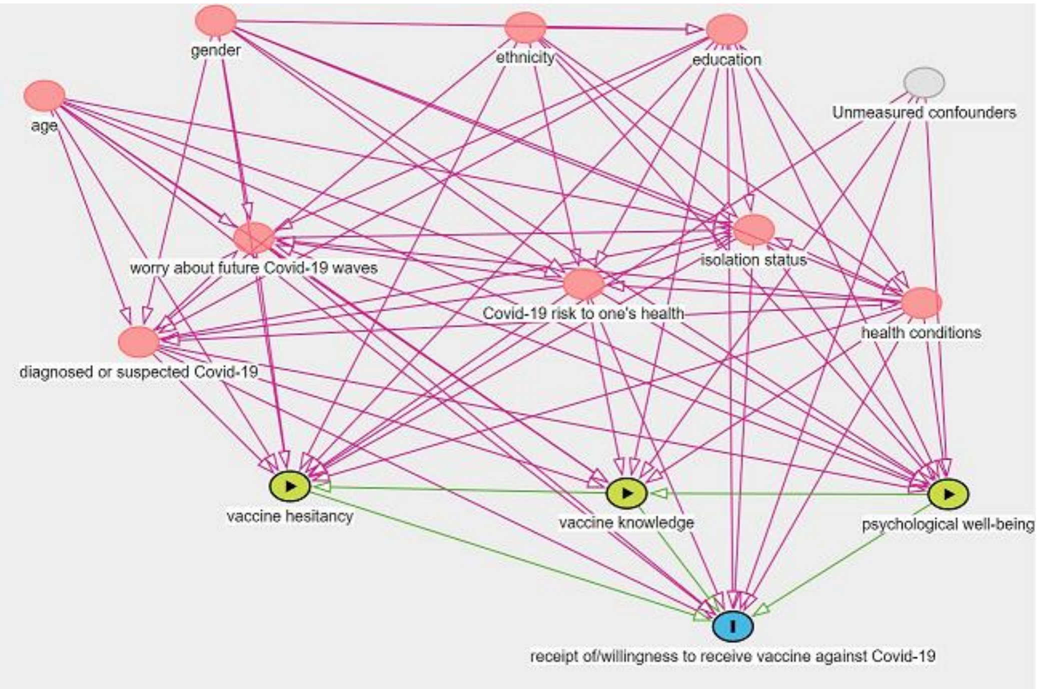

Supplementary figure 1. Directed acyclic graph (DAG) depicting which covariates should be included in a multivariable statistical model in order to minimise bias in the estimate of the total causal effect of i) vaccine hesitancy, ii) vaccine knowledge and iii) psychological well-being during the Covid-19 pandemic and (RQ1i) receipt of/willingness to receive a vaccine against Covid-19. The DAG was developed based on evidence from the existing literature and consensus within the research team.

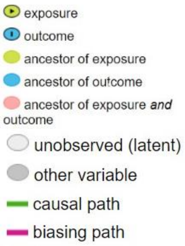

For peer review only - http://bmjopen.bmj.com/site/about/guidelines.xhtml

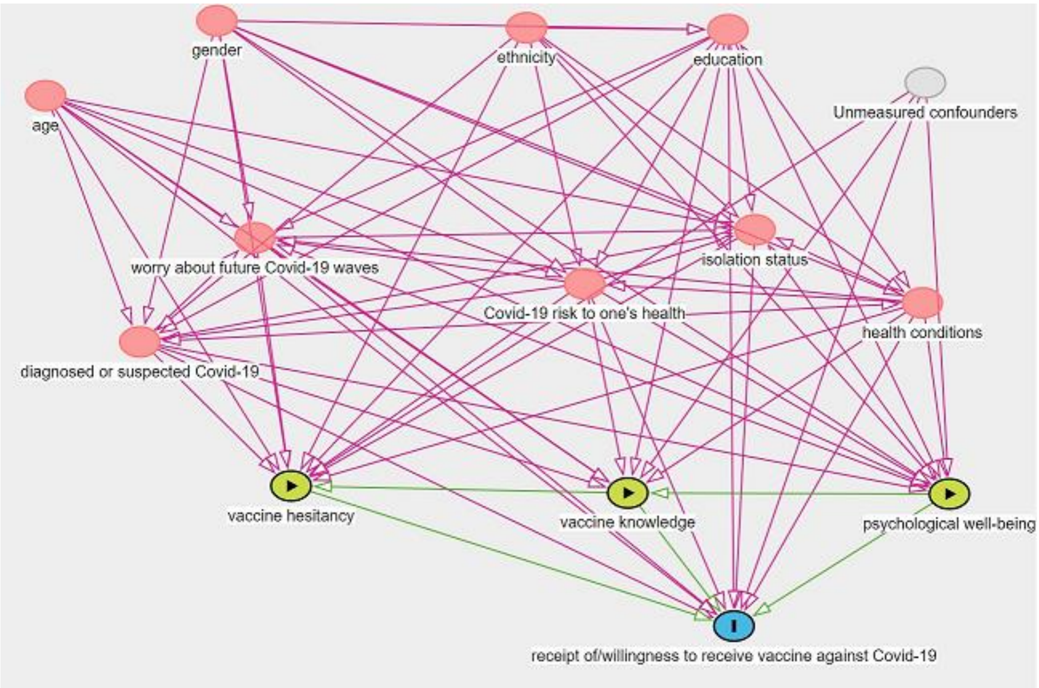

Supplementary figure 2. Directed acyclic graph (DAG) depicting which covariates should be included in a multivariable statistical model in order to minimise bias in the estimate of the total causal effect of i) vaccine hesitancy, ii) vaccine knowledge and iii) psychological well-being during the Covid-19 pandemic and (RQ2) willingness to get vaccinated against Covid-19 on a yearly basis. The DAG was developed based on evidence from the existing literature and consensus within the research team.

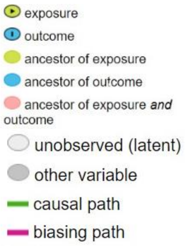

Supplementary table 1. Baseline characteristics of included and excluded sample (unweighted).

	**Total sample N=2,992**	**Included N=1,565 (52.3%)**	**Excluded N=1,427 (47.7%)**	***p***
Age in years M(SD)	47.92 (15.46)	51.74 (14.41)	43.74 (15.49)	<0.001
Female sex, % (N)	68.6 (2,054)	71.3 (1,114)	66.1 (940)	0.002
White ethnicity, % (N)	93.7 (2,804)	95.4 (1,487)	92.6 (1,317)	0.001
Post-16 qualifications, % (N)	86.7 (2,595)	88.9 (1,392)	84.3 (1,203)	<0.001
Health problems, % (N)	41.1 (1,208)	42.4 (659)	39.6 (549)	0.12

M=Mean, SD=Standard Deviation

For peer review only - http://bmjopen.bmj.com/site/about/guidelines.xhtml

Supplementary table 2. Associations between vaccine hesitancy and willingness to get vaccinated against Covid-19 on a yearly basis (sensitivity unweighted analysis).

	Willingness to get yearly vaccination against Covid-19 (reference category=yes)							
	No				Not sure			
	N=1,565		N=1,406		N=1,565		N=1,406	
	OR [95% CI]	*P*	aOR [95% CI]	*P*	OR [95% CI]	*P*	aOR [95% CI]	*P*
	N=1,565							
Vaccine hesitancy	147.70 [67.78-321.86]	<0.001	28.68 [14.38-57.20]	<0.001	17.89 [10.68-29.96]	<0.001	7.94 [4.48-14.08]	<0.001
Vaccine knowledge	0.27 [0.22-0.34]	<0.001	0.47 [0.36-0.62]	<0.001	0.42 [0.34-0.50]	<0.001	0.46 [0.36-0.59]	<0.001
Psychological well-being	0.69 [0.53-0.88]	0.003	1.07 [0.83-1.39]	0.596	0.74 [0.61-0.90]	0.002	0.94 [0.76-1.17]	0.580
*Health problems: yes	1 (ref)		1 (ref)		1 (ref)		1 (ref)	
No	1.30 [0.77-2.22]	0.330	1.35 [0.77-2.36]	0.290	0.85 [0.58-1.26]	0.416	0.82 [0.53-1.27]	0.372
Perceived high risk of Covid-19: no	1 (ref)		1 (ref)		1 (ref)		1 (ref)	
Yes	0.65 [0.23-1.82]	0.411	0.74 [0.28-1.94]	0.545	0.63 [0.29-1.37]	0.247	0.54 [0.24-1.20]	0.128
Confirmed/suspected Covid-19: no	1 (ref)		1 (ref)		1 (ref)		1 (ref)	
Yes	2.50 [1.49-4.17]	<0.001	1.52 [0.89-2.59]	0.122	1.61 [1.08-2.38]	0.022	1.07 [0.68-1.68]	0.773
Being in isolation: no	1 (ref)		1 (ref)		1 (ref)		1 (ref)	
Yes	4.76 [1.00-20.00]	0.049	3.60 [0.39-33.33]	0.261	1.27 [0.16-10.00]	0.821	2.21 [0.10-49.46]	0.618
**Receipt of/willingness to receive an initial Covid-19 vaccine: yes	1 (ref)		1 (ref)		1 (ref)		1 (ref)	
No	-		-		-		-	
Age (cont.)	0.99 [0.97-1.00]	0.087	0.98 [0.96-1.01]	0.123	0.98 [0.97-0.99]	0.002	0.98 [0.96-0.99]	0.005
Worry about future Covid-19 wave	0.96 [0.95-0.97]	<0.001	0.98 [0.97-0.99]	0.001	0.98 [0.97-0.99]	<0.001	1.00 [0.99-1.01]	0.436

For peer review only - http://bmjopen.bmj.com/site/about/guidelines.xhtml

Time of completion	1.03 [1.00-1.06]	0.032	1.04 [1.01-1.07]	0.021	1.01 [0.99-1.03]	0.480	1.00 [0.98-1.03]	0.821

OR=odds ratio; aOR=adjusted odds ratio; CI=Confidence Interval; ref=reference category
The reference category for the outcome variable was the response option 'yes'
*In unadjusted analysis of Health problems N=1,554.
** In unadjusted analysis of Receipt of/willingness to receive Covid-19 vaccine for first time N=1,415.

For peer review only

For peer review only - http://bmjopen.bmj.com/site/about/guidelines.xhtml

STROBE 2007 (v4) Statement—Checklist of items that should be included in reports of *cross-sectional studies*

Section/Topic	Item #	Recommendation	Reported on page #
**Title and abstract**	1	(*a*) Indicate the study's design with a commonly used term in the title or the abstract	1
		(*b*) Provide in the abstract an informative and balanced summary of what was done and what was found	2
**Introduction**			
Background/rationale	2	Explain the scientific background and rationale for the investigation being reported	4-7
Objectives	3	State specific objectives, including any prespecified hypotheses	7-8
**Methods**			
Study design	4	Present key elements of study design early in the paper	8
Setting	5	Describe the setting, locations, and relevant dates, including periods of recruitment, exposure, follow-up, and data collection	8-9
Participants	6	(*a*) Give the eligibility criteria, and the sources and methods of selection of participants	8-9
Variables	7	Clearly define all outcomes, exposures, predictors, potential confounders, and effect modifiers. Give diagnostic criteria, if applicable	9-12
Data sources/ measurement	8*	For each variable of interest, give sources of data and details of methods of assessment (measurement). Describe comparability of assessment methods if there is more than one group	9-12
Bias	9	Describe any efforts to address potential sources of bias	*9-12*
Study size	10	Explain how the study size was arrived at	9-12
Quantitative variables	11	Explain how quantitative variables were handled in the analyses. If applicable, describe which groupings were chosen and why	9-12
Statistical methods	12	(*a*) Describe all statistical methods, including those used to control for confounding	12-14
		(*b*) Describe any methods used to examine subgroups and interactions	12-14
		(*c*) Explain how missing data were addressed	12-14
		(*d*) If applicable, describe analytical methods taking account of sampling strategy	12-14
		(*e*) Describe any sensitivity analyses	10, 13
**Results**			

For peer review only

Participants	13*	(a) Report numbers of individuals at each stage of study—eg numbers potentially eligible, examined for eligibility, confirmed eligible, included in the study, completing follow-up, and analysed	14
		(b) Give reasons for non-participation at each stage	14
		(c) Consider use of a flow diagram	Not applicable
Descriptive data	14*	(a) Give characteristics of study participants (eg demographic, clinical, social) and information on exposures and potential confounders	14, table 1
		(b) Indicate number of participants with missing data for each variable of interest	14
Outcome data	15*	Report numbers of outcome events or summary measures	14-16
Main results	16	(*a*) Give unadjusted estimates and, if applicable, confounder-adjusted estimates and their precision (eg, 95% confidence interval). Make clear which confounders were adjusted for and why they were included	14-16
		(*b*) Report category boundaries when continuous variables were categorized	Not applicable
		(*c*) If relevant, consider translating estimates of relative risk into absolute risk for a meaningful time period	Not applicable
Other analyses	17	Report other analyses done—eg analyses of subgroups and interactions, and sensitivity analyses	14-16
**Discussion**			
Key results	18	Summarise key results with reference to study objectives	16-17
Limitations	19	Discuss limitations of the study, taking into account sources of potential bias or imprecision. Discuss both direction and magnitude of any potential bias	20-21
Interpretation	20	Give a cautious overall interpretation of results considering objectives, limitations, multiplicity of analyses, results from similar studies, and other relevant evidence	16-20
Generalisability	21	Discuss the generalisability (external validity) of the study results	16-20
**Other information**			
Funding	22	Give the source of funding and the role of the funders for the present study and, if applicable, for the original study on which the present article is based	23

*Give information separately for cases and controls in case-control studies and, if applicable, for exposed and unexposed groups in cohort and cross-sectional studies.

Note: An Explanation and Elaboration article discusses each checklist item and gives methodological background and published examples of transparent reporting. The STROBE checklist is best used in conjunction with this article (freely available on the Web sites of PLoS Medicine at http://www.plosmedicine.org/, Annals of Internal Medicine at http://www.annals.org/, and Epidemiology at http://www.epidem.com/). Information on the STROBE Initiative is available at www.strobe-statement.org.

For peer review only
